# Meeting the Unmet Needs of Individuals With Mental Disorders: Scoping Review on Peer-to-Peer Web-Based Interactions

**DOI:** 10.2196/36056

**Published:** 2022-12-05

**Authors:** Dawid Storman, Paweł Jemioło, Mateusz Jan Swierz, Zuzanna Sawiec, Ewa Antonowicz, Anna Prokop-Dorner, Marcelina Gotfryd-Burzyńska, Malgorzata M Bala

**Affiliations:** 1 Chair of Epidemiology and Preventive Medicine Department of Hygiene and Dietetics Jagiellonian University Medical College Krakow Poland; 2 AGH University of Science and Technology Krakow Poland; 3 Students’ Scientific Research Group of Systematic Reviews Jagiellonian University Medical College Krakow Poland; 4 Chair of Epidemiology and Preventive Medicine Jagiellonian University Medical College Krakow Poland; 5 Institute of Psychology Jagiellonian University Krakow Poland

**Keywords:** scoping review, peer-to-peer interactions, mental disorders, web-based interactions

## Abstract

**Background:**

An increasing number of online support groups are providing advice and information on topics related to mental health.

**Objective:**

This study aimed to investigate the needs that internet users meet through peer-to-peer interactions.

**Methods:**

A search of 4 databases was performed until August 15, 2022. Qualitative or mixed methods (ie, qualitative and quantitative) studies investigating interactions among internet users with mental disorders were included. The φ coefficient was used and machine learning techniques were applied to investigate the associations between the type of mental disorders and web-based interactions linked to seeking help or support.

**Results:**

Of the 13,098 identified records, 44 studies (analyzed in 54 study-disorder pairs) that assessed 82,091 users and 293,103 posts were included. The most frequent interactions were noted for people with eating disorders (14/54, 26%), depression (12/54, 22%), and psychoactive substance use disorders (9/54, 17%). We grouped interactions between users into 42 codes, with the *empathy or compassion* code being the most common (41/54, 76%). The most frequently coexisting codes were *request for information* and *network* (35 times; φ=0.5; *P*<.001). The algorithms that provided the best accuracy in classifying disorders by interactions were decision trees (44/54, 81%) and logistic regression (40/54, 74%). The included studies were of moderate quality.

**Conclusions:**

People with mental disorders mostly use the internet to seek support, find answers to their questions, and chat. The results of this analysis should be interpreted as a proof of concept. More data on web-based interactions among these people might help apply machine learning methods to develop a tool that might facilitate screening or even support mental health assessment.

## Introduction

### Background

It is estimated that 38.2% of Europeans and 26.2% of Americans experience mental disorders annually [1,2]. Unfortunately, social perception of these disorders is largely based on stereotypes [3]. Despite antistigma campaigns [4], stigmatization and discriminatory practices are reinforced by media discourse that reproduces false and simplified mental representations of people with mental disorders [5-8]. Therefore, many individuals seek information or support on the web. The internet is an essential platform for creating web-based communities that provide a venue to ask questions, share experiences, and offer mutual emotional support [9]. Most studies reporting evidence that websites can provide meaningful help focused on people with physical disorders such as cancer [10], diabetes mellitus [11], and Alzheimer disease [12]. However, research suggests that more attention should be paid to people with psychological conditions, including those who self-harm [13], those who experience eating disorders [14], and those with various other mental disorders [15] because these conditions affect different aspects of daily functioning. According to the literature, people with mental disorders are willing to connect with others using social media [9] even though they have greater difficulties in establishing relationships offline than people without such disorders [16]. This trend was further reinforced during the COVID-19 pandemic when access to face-to-face professional help became limited and was replaced by remote support services [17]. At the same time, the number of internet users grew from 4.1 billion in 2019 to 4.9 billion in 2021 [18], which means that a higher number of people could benefit from our research.

According to several studies, both people who generate content and those who interact with creators may benefit from such an interaction [19-21]. Unlike spontaneous offline meetings, web-based interactions do not require the same level of engagement or instant reactions. Thus, this type of interactions may help people with mental disorders overcome increased levels of social anxiety or face information-processing challenges [22]. This, in turn, may provide a sense of empowerment and lead to shorter recovery times. In addition, the internet can offer anonymity, making web-based interactions with strangers less threatening than in-person contact [23].

Self-esteem is built on several key factors, one of which is a sense of belonging to a group [24]. Therefore, connecting with similar individuals (peers) may result in better recovery and social integration among people with mental disorders [25]. However, stigmatization and rejection can happen even within the communities themselves [26] but also in web-based interactions. Thus, it is critical for internet users with mental disorders to join the right web-based groups to avoid rejection from their peers. Unfortunately, there are still an insufficient number of mental health professionals who can provide necessary assistance within a web-based community. Therefore, internet users become organized into self-help groups. Available evidence demonstrates that web-based interactions between peers have enormous potential to help bridge the gap between the identified need for services and the limited resources available for conventional treatment [27].

A peer is defined as a person who has the same social position or abilities as other members of a group [28]. There are several types of peer relationships, such as (1) between a peer and another individual (dyad), (2) between a peer and a group, and (3) a hybrid of both types [29,30]. Furthermore, the types of peer-to-peer interactions are heterogeneous and may include mutual support or participation in consumer assistance or peer-run programs [25,31]. Some of these can occur web-based via different platforms available, such as support groups, forums, discussion groups, bulletin boards, social media, and chats [32].

Peer-to-peer interactions allow people to share experiences, exchange information, and provide advice and emotional support in a natural and spontaneous manner. Therefore, they constitute an exciting subject of research. There is evidence showing that relationships between peers promote behavioral changes [33], improve coping strategies [34], and alleviate social isolation and loneliness among people with mental disorders [35-37]. For many people, social networking on the internet is the major form of communication that facilitates social interactions [38]. This is especially true for individuals who experience difficulties in direct contact with others because of stigmatization [39]. Barak et al [40] reported lower levels of emotional distress among adolescents when they were involved in a web-based forum. However, peer-to-peer interactions on the internet may also negatively affect mental health. Generally, internet use raises concerns, such as user behavior control, accurate risk assessment, privacy, and confidentiality [41].

Currently, new technologies are being developed for people with mental disorders, including artificial intelligence (AI) that already plays a major role in general medicine and research [42-44]. Techniques based on AI are widely applied in medical imaging diagnostics [45-47], but they can also be used for personalization purposes [48-50]. By identifying patterns in the types of interactions linked to specific types of disorders, these techniques could help individualize interventions provided by moderators of web-based forums. AI might also serve as a supporting tool in situations where there are no forum administrators (eg, owing to high costs). It can tailor the content to individual needs and concerns of the users.

### Objectives

So far, studies assessing peer-to-peer interactions, including systematic reviews [51-53], have focused on the efficacy of such interactions. However, studies that summarize qualitative research are lacking. To fill this gap, we conducted a scoping review that addressed the following research questions:

1. What are the needs that individuals with mental disorders fulfill through web-based peer-to-peer interactions?
2. What are the categories of peer-to-peer interactions and how can they be used in further research?
3. Is it possible to use machine learning (ML) techniques to assess and classify mental disorders based on the types of peer-to-peer interactions?

In our opinion, heterogeneous and multidimensional data can be best handled using ML techniques (or even deep learning if sufficient data are available). Therefore, the aim of this proof-of-concept study was to explore the potential of ML in such an analysis.

## Methods

The study was conducted in accordance with the PRISMA-ScR (Preferred Reporting Items for Systematic Reviews and Meta-Analyses extension for Scoping Reviews; Multimedia Appendix 1 [54,55]). The study protocol was registered in the Open Science Framework (added on August 24, 2020, and registered on November 11, 2020 [56]).

### Eligibility Criteria

For this analysis, we considered studies performed according to qualitative or mixed (ie, qualitative and quantitative) methodology that evaluated the following: (1) web-based interactions between participants with any mental disorder that is defined according to any standard diagnostic criteria and (2) interactions between a peer and another individual (dyad) [57]. Studies that assessed only family members or caregivers of people with mental disorders were excluded. No language or date restrictions were applied. In addition, the eligibility criteria were not limited to a specific location, publication status, or any other characteristic.

### Search Strategy

We searched 4 electronic databases (Ovid MEDLINE, Embase, Cochrane Library, and Web of Science) until August 15, 2022. The search was performed without any restrictions on the language or publication date of the studies. All search strategies are available in Multimedia Appendix 2. For additional papers, we manually searched the references of reviews that were obtained through the search.

### Study Selection

To identify eligible studies, titles, abstracts, and full texts were individually assessed by any 2 of the 5 reviewers (DS, PJ, MJS, MG, and APD). Conflicts were resolved by discussion or involvement of a third reviewer (DS or MMB).

### Data Charting

Data charting was performed independently by 2 of the 5 authors (DS, PJ, MJS, ZS, and EA). Disagreements were resolved by consensus or arbitration by a third reviewer (DS). All relevant data on research characteristics (eg, study design, country of origin, and funder), methodology (eg, type of coding and coding scheme), participants (eg, age, gender, and type of mental disorder), and results (interactions) were extracted since November 8, 2020.

### Credibility Assessment

The study quality was assessed by 2 of the 4 independent reviewers (MJS, EA, ZS, and PJ) using the Critical Appraisal Skills Programme (CASP) checklist for qualitative research [58]. The tool included 10 questions about study validity, study results, and whether the results helped locally. They could be answered by selecting *yes*, *no*, or *can’t tell*. We divided the final question (*How valuable is the research?*) into 3 subquestions according to the hints provided in the manual: (1) input into existing knowledge (10a); (2) identification of unexplored areas (10b); and (3) external validity of the findings (10c). These 3 criteria were scored as 0 (not fully met) or 1 (fully met). The general assessment of the study quality was based on the sum of the scores from the 3 subquestions. A score of 3 indicated a valuable study; 2, a moderately valuable study; 1, a study of some quality; and 0, a study of no quality. Any disagreements were resolved by the involvement of a third independent reviewer (DS).

### Synthesis of Results

The essential data on the population and methodology of the included studies were summarized in tabular and descriptive forms. All types of interactions observed in the studies were grouped into several categories (codes), which were defined based on the previous literature. To describe the categories and the links between them, several models were used (both originally developed and derived from the literature). The models were created during the discussion between the coauthors (DS, PJ, and APD), and they were presented as partition trees (Multimedia Appendix 3). The models were evaluated based on the lowest SD value of the number of codes in the category, which was the most common measure of the dispersion of results [59]. The frequency of codes as well as the co-occurrence of codes and diseases were presented using heatmaps (means and sums) and a circular chart (co-occurrence frequencies) to investigate possible associations between interactions and specific disorders.

All graphs were prepared using Python 3.7.10 (Python Software Foundation) libraries: Matplotlib 3.2.2 (John Hunter), Seaborn 0.11.1 (Michael Waskom), NetworkX 2.5.1 (Aric Hagberg, Dan Schult and Pieter Swart), Graphviz 2.47.1 (John Ellson), VOSviewer 1.6.6 (Nees Jan van Eck and Ludo Waltman), or Microsoft Office 2004 (Microsoft Corp). The source code is available on GitHub.

### Statistical Analysis

We used the φ coefficient [60] to examine the associations between the types of interactions and mental disorders. Using Pandas 1.1.5 (Wes McKinney) and NumPy 1.19.5 (Travis Oliphant), we represented the data as a data frame and then used SciPy 1.4.1 (Travis Oliphant, Pearu Peterson, and Eric Jones) to calculate associations and their statistical significance.

Associations within the following subgroups were evaluated: (1) type of disorder, (2) studies assessed as valuable versus other studies, and (3) types of disorder using only valuable (high-quality) studies. ML techniques were applied to classify mental disorders based on interactions between users. For this purpose, several basic algorithms were used. These algorithms were selected based on their strong mathematical background and resultant explainability properties, as we were interested in identifying the variables that contributed to performance [61]. More specifically, we incorporated decision trees (with minimum samples per leaf ranging from 1 to 3), logistic regression (with L2 regularization), support vector machines (with the radial basis function kernel), k-nearest neighbors algorithm (with k ranging from 2 to 5), and Gaussian naïve Bayes classifier (default settings). For this analysis, Scikit-learn (version 0.22.2) was used.

### Mapping the Terms

To examine the relations between the terms as well as construct and visualize bibliometric networks, we used the mapping software VOSviewer (version 1.6.16) [62]. We aimed to investigate the co-occurrence networks of important terms extracted from the full text of the included studies. Thus, we provided a visualization. The distance between any pair of objects reflects their similarity as accurately as possible. Objects with high similarity are located close to each other, whereas objects with low similarity are located far from each other. We created a co-occurrence map by applying the default counting method and choosing number 5 as the minimum number of occurrences considering the most advantageous setting in terms of resources, time, and data received [62]. A total of 2 independent reviewers (DS and PJ) screened the list of terms extracted from VOSviewer and selected those that described the interactions. Any discrepancies were resolved through discussion. The final terms were used to create visualizations. In addition, we compared the terms selected from VOSviewer with the codes from our codebook and calculated the percentage of overlap.

## Results

### Overview

**Figure 1:**
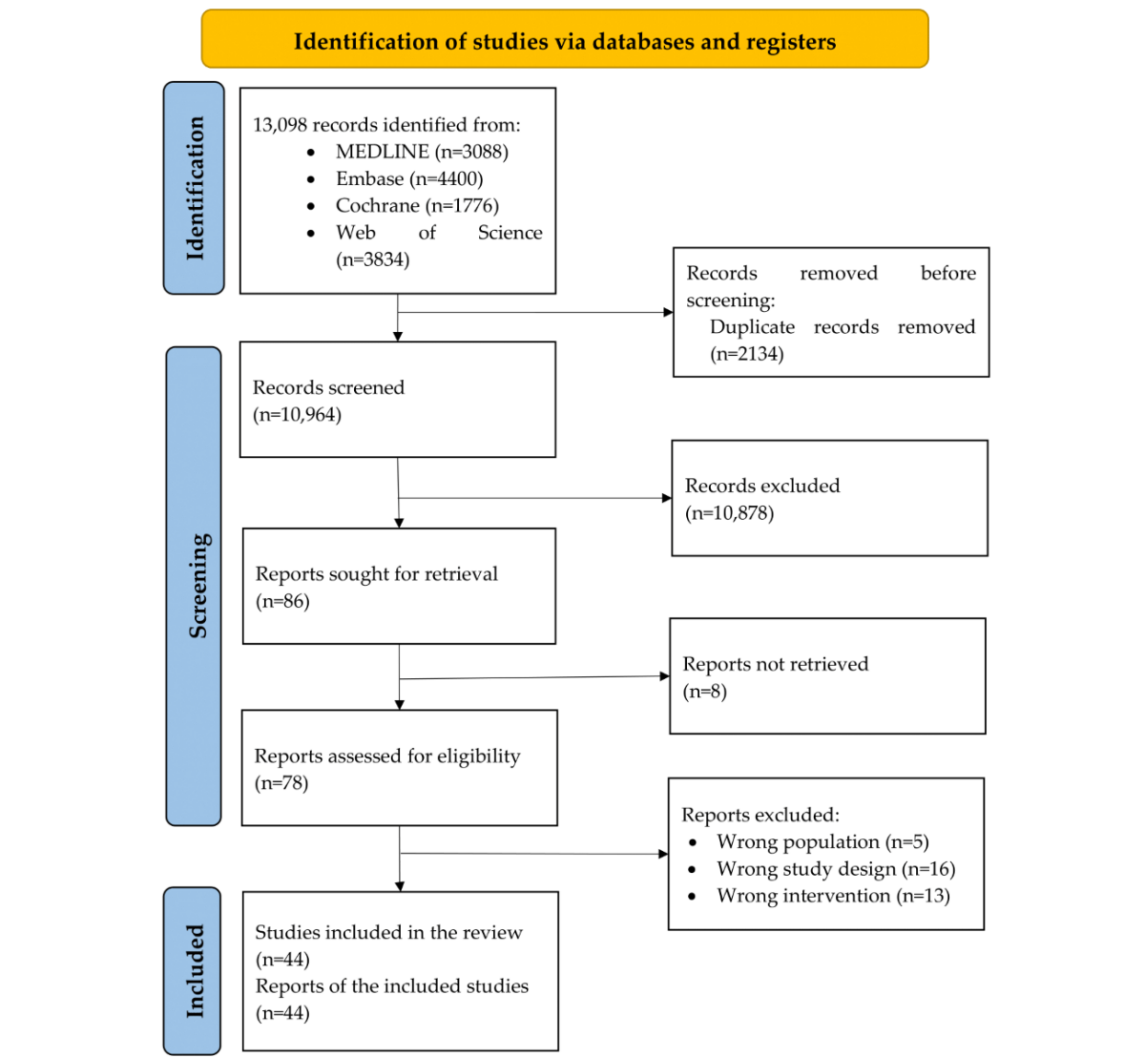
PRISMA (Preferred Reporting Items for Systematic Reviews and Meta-Analyses) flow diagram.

The search identified 13,098 original references, and the screening of titles and abstracts yielded 86 full-text papers. A total of 44 studies were included in the final analysis and 8 were labeled as ongoing (Multimedia Appendix 4 [63-105] and Multimedia Appendix 5 [104-111]). The study flow is presented as a PRISMA (Preferred Reporting Items for Systematic Reviews and Meta-Analyses) flow diagram in Figure 1 [112]. A list of excluded studies with the reason for exclusion is provided in Multimedia Appendix 6 [113-146].

### Included Studies

The detailed characteristics of the 44 included studies (analyzed in 54 study-disorder pairs) are presented in Table 1 and Multimedia Appendix 7 [63-105]. These studies were conducted between the years 2000 and 2022. Among the corresponding authors, 82% (36/44) originated from English-speaking countries, including the United States (16/44, 36%), the United Kingdom (9/44, 20%), Canada (6/44, 14%), Australia (2/44, 5%), Ireland (1/44, 2%), Singapore (1/44, 2%), and Hong Kong (1/44, 2%); whereas 18% (8/44) originated from non–English-speaking countries, including Sweden (3/44, 7%), Israel (2/44, 5%), Switzerland (1/44, 2%), Italy (1/44, 2%), and Hungary (1/44, 2%). None of the included studies provided information on whether the study protocol was registered in an appropriate registry.

**Table 1:** Characteristics of the included studies (N=44).

Variable			Value, n (%)^a^
**Place of interaction**			
	Forum	26 (59)	
	Media (Facebook, Instagram, etc)	12 (27)	
	Support group	7 (16)	
	Blog	1 (2)	
	Chat	1 (2)	
**Access to the place of interaction**			
	Public access	25 (57)	
	Registration	4 (9)	
	Partial access (need to register to add comments)	6 (14)	
	Not reported	10 (23)	
**Type of analysis used**			
	Content analysis	23 (52)	
	Thematic analysis	13 (30)	
	Discourse analysis	5 (11)	
	Constant comparison	2 (5)	
	Conversational analysis	2 (5)	
	Other	13 (30)	
	Not reported	1 (2)	
**Coding schemes for social support and interaction**			
	Cutrona and Suhr	5 (11)	
	The self-reported coding scheme	3 (7)	
	Other	7 (16)	
	Not reported	33 (75)	

^a^Some studies could be included in >1 subgroup.

### Participants

The studies included 82,091 users (mean 3284, SD 9526; range 11-41,967) who posted 293,106 comments (mean 8374, SD 25,105; range 4-132,599) on 19,940 topics (mean 1173, SD 2916; range 5-10,169). The age of the participants ranged from 16 to 78 years, although most studies (37/44, 84%) did not report data on age. The proportion of women ranged from 0% to 100% (mean 65%, SD 34%); however, most studies (31/44, 70%) did not provide this information. The percentage of inactive to passive forum users was 47.62% (range 17%-85.47%). In 18% (8/44) of studies, participants were recruited to register on a forum created by the authors themselves.

There were 22 different mental disorders classified into 13 categories. Most commonly, studies have assessed peer-to-peer interactions among individuals with eating disorders (14/54, 26%), depression (12/54, 22%), and psychoactive substance use disorders (9/54, 17%). Next, there were the following disorders: postpartum depression (4/54, 7%), anxiety disorders (3/54, 6%), posttraumatic stress disorder (2/54, 4%). The remain disorders occurred only once (1/54, 2%): attention-deficit/hyperactivity disorder, bipolar affective disorder, mild cognitive impairment, obsessive compulsive disorder, schizoaffective disorder, and schizophrenia.

### Type of Platform for Interaction

Of the 44 platforms, 11 (25%) platforms were developed specifically for the mental health setting, 3 (7%) platforms were intended for more general health, and for 30 (68%) platforms, this information was not reported. Most studies assessed web-based forum interactions (26/44, 59%). In most cases, access to the place of interaction was free and registration was not required to add comments (25/44, 57%). However, in some cases, registration was required to add and read comments (4/44, 9%). However, in some other cases, forums offered partially free access, with registration required to add, but not read, comments (6/44, 14%).

Most studies reported the presence of moderators (22/44, 50%). Their roles included the following: (1) provision of advice or therapy, (2) monitoring and control of content (sensitive, legal, sexual, abuse, and eliminate spam), or (3) moderation of discussions.

Anonymity was ensured in most of the studies (35/44, 80%). The authors paraphrased participants’ statements and comments, did not record any personal data, excluded nicknames from the analysis, replaced nicknames with initials, or did not include quotations in the text. Membership terms of use were specified in 36% (16/44) of studies. For example, by accepting the terms and conditions, users agreed to treat other members with respect, provide support, avoid profanity and unhelpful language, avoid detailed and vivid descriptions of self-harming techniques, not offer drugs to other members, and provide links to sites selling drugs.

### Methods in the Included Studies

Of the 44 studies, 17 (39%) studies used only qualitative methodology, whereas 19 (43%) studies also used a frequency analysis. Mixed methods (ie, qualitative and quantitative) were applied in 18% (8/44) of studies.

Content and thematic analyses were the most common (23/44, 52% and 13/44, 30%, respectively). Other analyses included membership categorization, ethnomethodological, netnographic, rhetorical, framework, interpretative phenomenological, image, sequence, paths, and social networks, each applied in a single study.

A low level of precision regarding reporting on methodological approaches impeded comparisons of the analytic strategies used by the authors. Coding performed by 2 people was reported in 52% (23/44) of studies. In 27% (12/44) of studies, complete coding was performed independently, in 16% (7/44) of studies, it was performed only during the calibration process, and in 9% (4/44) of studies, one author coded part of the material and the other author checked that coding. Coding was applied inductively in 43% (19/44) of studies and deductively in 14% (6/44) of studies. In 11% (5/44) of studies, both approaches were used, and the remaining 32% (14/44) of studies did not report the coding strategy. A codebook was developed openly (without blinding) in 18% (8/44) of studies. Blinding was established in 2% (1/44) of papers. The coding scheme for social support designed by Cutrona and Suhr [147] was used in 11% (5/44) of studies, whereas 16% (7/44) of studies adopted different approaches proposed by Cohen and Wills [148], Oakley [149], Tong et al [150], Bauer et al [151], Morse and Field [152], Gaysynsky et al [153], and Bales [57]. In 7% (3/44) of studies, the authors used their own system [63,154,155].

To determine interrater agreement, Cohen κ was used in 14% (6/44) of studies, Krippendorff α was used in 2% (1/44), and data were not reported in the remaining 84% (37/44) of papers. In 4 studies, the diagnosis of participants was confirmed using the Center for Epidemiologic Studies Depression Scale or by a specialist.

### Types of Interactions

**Figure 2:**
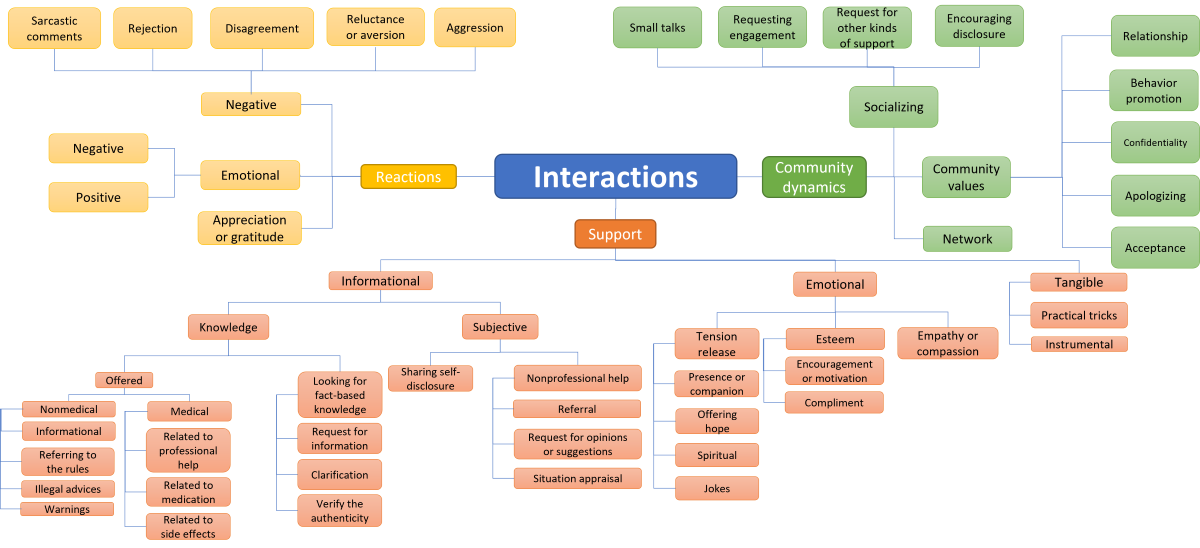
Categorization of peer-to-peer interactions.

We distinguished 42 codes that described peer-to-peer interactions. The codes were organized into 15 categories from A to O (Table 2), and we proposed 14 different models of coding interventions among peers (Multimedia Appendix 3). Six models were based on existing models: 1 [147], 3 [156], 4 [153], 5 (adapted from Liu et al [157]), 6 [158], and 7 [57]. A total of 2 models were modified based on the models by Cutrona and Suhr [147] (model 2) and Greiner et al [64] (model 8). The remaining 6 models were developed by us (models 9, 10, 11, 12, 13, and 14). After calculating the means and SDs, the models were ranked based on the lowest SD (Multimedia Appendix 8). Model 14 was characterized by the lowest SD (9.32), and the corresponding tree is presented in Figure 2.

**Figure 3:**
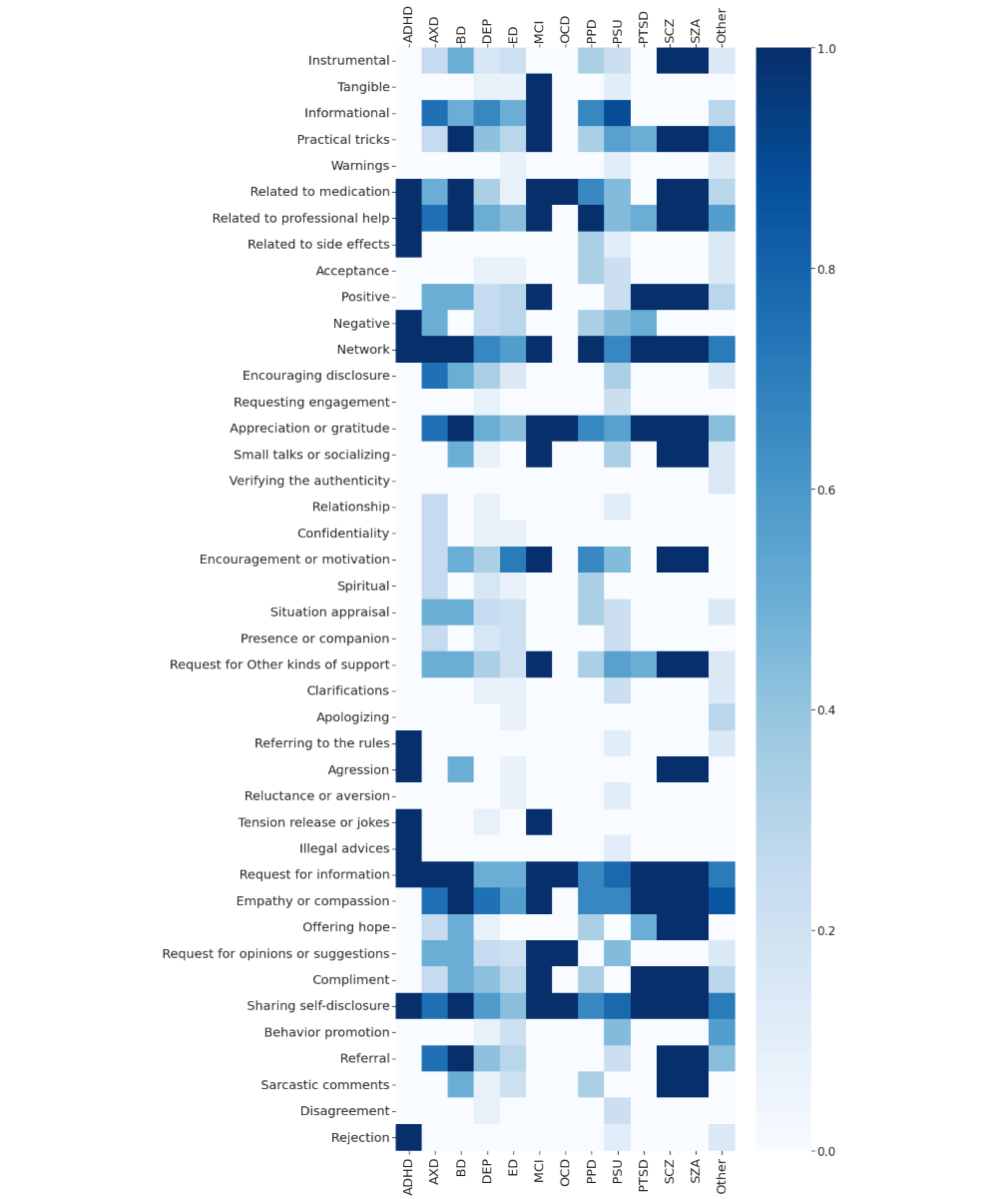
Heatmap with normalized means of code co-occurrence among mental disorders. ADHD: attention-deficit/hyperactivity disorder; AXD: anxiety disorders; BD: bipolar affective disorder; DEP: depression; ED: eating disorders; MCI: mild cognitive impairment; OCD: obsessive compulsive disorder; PPD: postpartum depression; PSU: psychoactive substance use; PTSD: posttraumatic stress disorder; SCZ: schizophrenia; SZA: schizoaffective disorder.

The most frequent interactions were *empathy or compassion* (41/54, 76%), *network* (40/54, 74%), and *sharing self-disclosure* (39/54, 72%). Heatmaps of the selected codes and disorders and their co-occurrence are presented in Figure 3 and Multimedia Appendices 9 and 10. We visualized the normalized means of code co-occurrence across disorders and all included studies. However, heatmaps should be interpreted with caution because of the unequal number of papers regarding individual disorders, which resulted in certain codes being used significantly more often.

**Figure 4:**
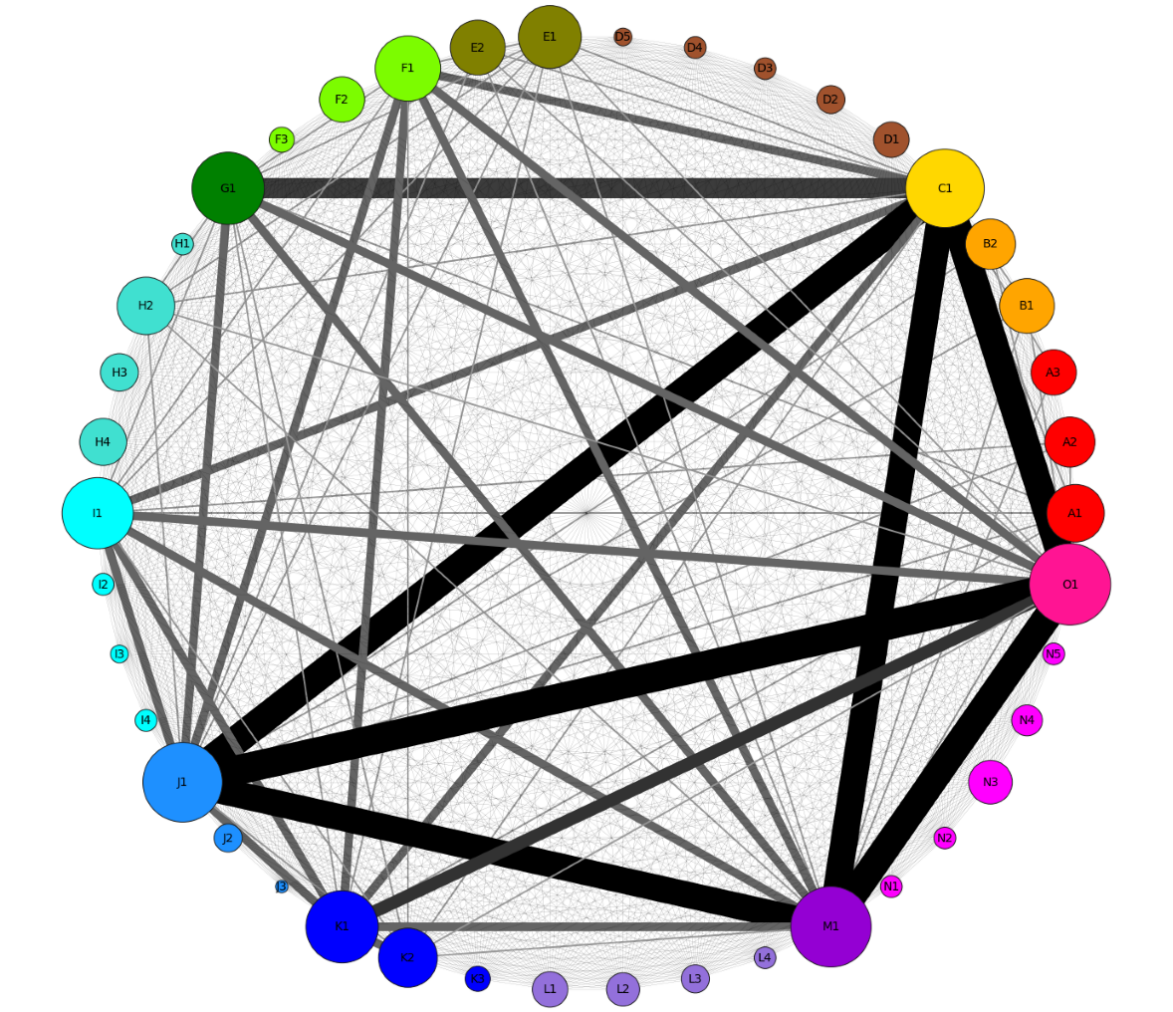
Circular chart of the co-occurrence of codes. The nodes represent specific types of interaction (Table 2). Node size corresponds to the number of primary studies that mention this type of interaction. The edges of the graphs indicate co-occurrence with other codes, and their size is proportional to the number of co-occurrences. A1: referral; A2: request for opinions or suggestions; A3: situation appraisal; B1: positive; B2: negative; C1: sharing self-disclosure; D1: sarcastic comments; D2: aggression; D3: disagreement; D4: rejection; D5: reluctance or aversion; E1: encouragement or motivation; E2: compliment; F1: practical tricks; F2: instrumental; F3: tangible; G1: appreciation or gratitude; H1: requesting engagement; H2: request for other kinds of support; H3: small talks or socializing; H4: encouraging disclosure; I1: informational; I2: referring to the rules; I3: illegal advice; I4: warnings; J1: request for information; J2: clarifications; J3: verifying the authenticity; K1: related to professional help; K2: related to medication; K3: related to side effects; L1: presence or companions; L2: offering hope; L3: spiritual; L4: tension release or jokes; M1: empathy or compassion; N1: apologizing; N2: confidentiality; N3: behavior promotion; N4: acceptance; N5: relationship; O1: network.

The co-occurrence of all codes is shown in Figure 4. Co-occurrence was observed most often for *request for information* and *network* (35 times). There was a positive association between these 2 codes (φ=0.5; *P*<.001). The strongest positive association was noted between *requesting engagement* and *disagreement*, *relationship* and *confidentiality*, and *referring to the rules* and *rejection* (φ=0.65; *P*<.001). As for correlations between codes and disorders, the strongest correlation was observed between attention-deficit/hyperactivity disorder and *illegal advice* (φ=0.70; *P*<.001). The remaining associations for the overall and subgroup analyses are presented in Multimedia Appendix 10.

We achieved the highest accuracy in classifying disorders by interactions using 2 methods: decision trees (44/54, 81%) and logistic regression (40/54, 74%). The confusion matrices (with absolute values and relative percentages) of the ML techniques with detailed results are presented in Multimedia Appendix 11. In addition, using decision trees, we visualized the possible pathways to identify mental disorders (Multimedia Appendix 12).

**Table 2:** Codebook.

Node	Codes	Meaning
A1	Referral	Referring the recipient to other sources of information or help, other places in general, and nonprofessional Providing the recipient with access to new people or other communication channels
A2	Request for opinions or suggestions	Asking about any act that offers direction or action for how to engage in the task or advances a belief or the value that is relevant to the task
A3	Situation appraisal	Helping reassess or redefine the situation faced by the recipient
B1	Positive	Showing positive emotions
B2	Negative	Showing negative emotions
C1	Sharing self-disclosure	Speaking about oneself, one’s experience, and one’s disease (recovery reports, treatment, diagnosis, etc)
D1	Sarcastic comments	Being disrespectful, insolent toward other members or statements that express being hurt
D2	Aggression	Presenting hostile or violent attitudes toward another with or without readiness to attack or confront
D3	Disagreement	Expressing a different opinion
D4	Rejection	Expressing little desire to include a person in their groups and relationships or excluding a person
D5	Reluctance or aversion	Expressing a strong dislike or disinclination
E1	Encouragement or motivation	Providing the recipient with a motive for doing something and confidence
E2	Compliment	Improving one’s self-worth by saying positive things about the recipient
F1	Practical tricks	Sharing advice (not necessarily based on facts and can be based on self-experience) Providing ideas or suggestions for action
F2	Instrumental	Offering help or a talk
F3	Tangible	Sharing goods or services
G1	Appreciation or gratitude	Expressing appreciation to another individual from the group or the group all in all
H1	Requesting engagement	Asking for opportunity to participate or be involved in group’s life
H2	Request for other kinds of support	Asking about anything other than facts, opinions, or suggestions
H3	Small talks or socializing	Greetings Taking politely about unimportant or uncontroversial matters
H4	Encouraging disclosure	Motivating to expose oneself, revealing information about oneself
I1	Informational	Sharing information or theoretical knowledge (should be based on facts)
I2	Referring to the rules	Mentioning and enforcing the applicable group norms and rules
I3	Illegal advice	Mostly related to drugs—providing information about where one can buy drugs and how to deal with getting a prescription from a physician
I4	Warnings	Indicating a possible danger, problem, or other unpleasant situation
J1	Request for information	Asking questions to obtain an answer about facts
J2	Clarifications	Asking to make a statement or situation less confusing and more comprehensible (eg, asking for explanation or asking additional questions)
J3	Verifying the authenticity	Asking about proofs (eg, a code of diagnosis)
K1	Related to professional help	Providing information about places where one can obtain help from specialists
K2	Related to medication	Providing information about drugs, doses, and route of administration
K3	Related to side effects	Providing information about an unpleasant effect of a drug that occurs in addition to the main effect
L1	Presence or companions	Offering to be there
L2	Offering hope	Providing the recipient with hope
L3	Spiritual	Offering prayer for the recipient
L4	Tension Release or jokes	Posting messages that include humor Reducing the anxiety that a person or a group may be experiencing
M1	Empathy or compassion	Showing that their feelings are seen
N1	Apologizing	Expressing regret for doing something wrong
N2	Confidentiality	Keeping the recipient’s problem in confidence
N3	Behavior promotion	Supportive of harmful behavior Supportive of minimizing harmful behavior Unsupportive of harmful behavior
N4	Acceptance	Being received and admitted into a group
N5	Relationship	Conveying the importance of closeness
O1	Network	Providing agreement with the views of the recipient Providing validation, normalizing the situation Showing the problem or situation as affecting more people, helping in identification, solidarity, and group cohesion Alleviating any feelings of guilt that the recipient may have about the situation

### Quality of the Included Studies

**Figure 5:**
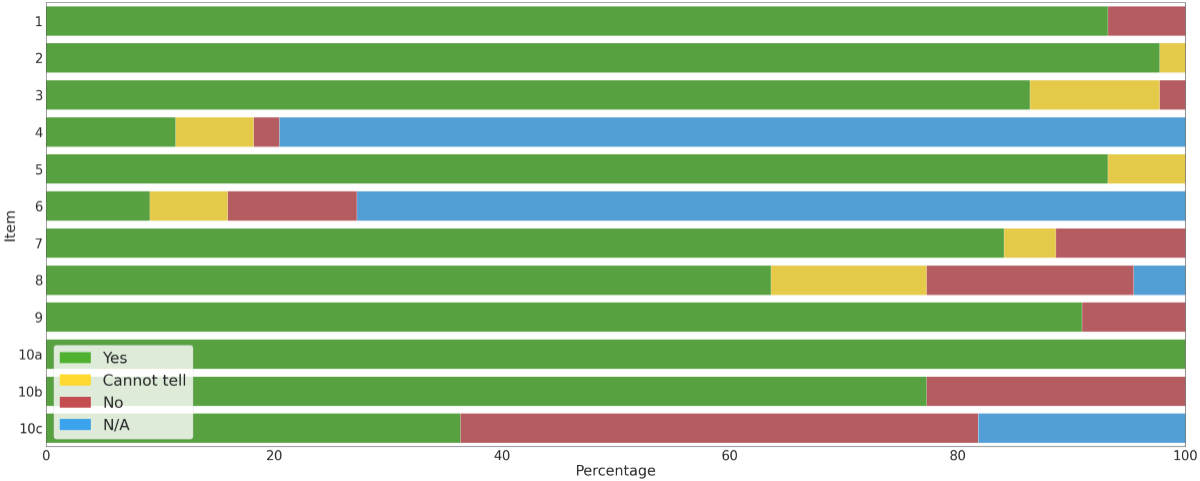
Overview of reviewer judgments on each Critical Appraisal Skills Programme (CASP) item across all studies. N/A: not applicable.

A detailed credibility assessment of the individual studies is presented in the Multimedia Appendix 13 [63-105]. An overview of reviewer judgments for each CASP item across all the studies is presented in Figure 5. Of the 44 studies, 13 (30%) studies were assessed as *valuable*; 24 (55%), as *moderately valuable*; and 7 (16%), as being *of some value*. All (44/44, 100%) the studies assessed in this review used an appropriate qualitative methodology and discussed the contribution of the included studies to existing knowledge. The weakest domain included discussing the applicability of the results to other populations or considering other uses for research (16/44, 36%). The mean quality score was 2.14 (SD 0.67), which corresponded to *moderately valuable* or *valuable* studies.

### Mapping the Terms

**Figure 6:**
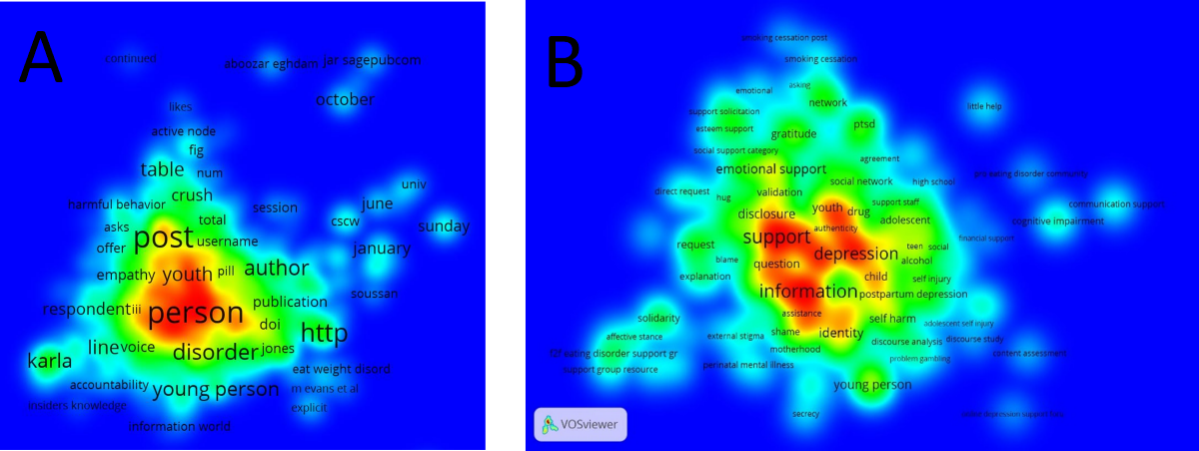
VOSviewer heatmaps of terms; (A) a heatmap of 2068 terms and (B) a heatmap of selected 345 terms.

Of the 2068 terms extracted from full-text articles (collected in 18 clusters; Figure 6A), we selected 345 (occurring 11,039 times in included papers) to create a term co-occurrence map (Figure 6B). It produced 15 clusters, which are listed in Multimedia Appendix 14, along with a list of terms. The most frequent terms related to peer-to-peer interactions were support (629/11,039, 5.69%), information (576/11,039, 5.22%), and experience (372/11,039, 3.37%). The overlap of terms describing interactions identified using VOSviewer with codes from our codebook was 96.3% (180/187). Some (7/187, 3.7%) categories did not have an equivalent in the code (*avoidance*, *blame*, *competition*, *discrimination*, *shame*, *tolerance*, and *trust*).

## Discussion

### Principal Findings

This systematic scoping review summarizes 44 studies that assessed peer-to-peer interactions among people with 22 different mental disorders. The interactions were categorized into 13 groups. The most common interactions such as *empathy or compassion*, *networking*, and *sharing self-disclosure* were observed on forums for people with eating disorders (14/54, 26%), depression (12/54, 22%), and psychoactive substance use (9/54, 17%). In this study, we focused on developing a codebook for future research. We believed that the reinterpretation of the data reported by the authors of the primary studies included in our review may have introduced bias. Therefore, we did not deliberately dwell on the coexistence of codes and specific disorders. For example, it seemed that sarcastic comments were present in most studies on schizophrenia and schizoaffective disorders. We could hypothesize that because of their condition, these people may be survivors of verbal aggression from their peers. However, it is possible that people with schizophrenia and schizoaffective disorders post sarcastic comments. Thus, this conclusion cannot be fully justified without looking into the primary data, but such an analysis was not the objective of this study. Nevertheless, the normalized means of code co-occurrence across disorders presented in Figure 3 are a good starting point to formulate hypotheses for our future primary studies.

Our study revealed the needs that prompt users to express themselves on the internet. For example, these may be information, emotional, or instrumental support needs. However, without primary research, it is difficult to determine whether these needs are fully (if at all) satisfied by internet use. We can assume that the unmet need is fulfilled by another internet user; however, these interactions will be the subject of future research. We are aware that because a systematic scoping review aims to map and identify gaps in current knowledge, we will generate more questions than answers. Nevertheless, we hope that this study will inspire future qualitative research in the field.

### Overall Completeness and Quality of Evidence

Our study provides evidence on the involvement of people with different mental disorders in online support groups. However, these disorders did not include personality disorders, organic mental disorders, or phobias, and there were no data on the experience of these individuals in seeking support on the internet. In addition, from the analysis of various interaction models, we noticed that some of the codes were not represented in our results because they might not have been assessed or the authors might have failed to report them (eg, owing to insufficient sample size). Therefore, we could not generalize the results to other populations because they may not fully reflect reality. As for the applied qualitative methodology, the authors used different approaches and analytical models. Owing to the diversity of theoretical perspectives, epistemological assumptions, and principles of conducting research, it can be challenging to apply a qualitative approach, including the comparison and synthesis of methods used in qualitative research [159]. Some authors did not comply with the available reporting guidelines [160-162]. Therefore, many studies lacked information on methodology, population characteristics, and outcomes.

Considering the level of adherence to the methodology and the applied methods themselves, we assessed the overall quality of the studies included in our analysis as moderate.

### Codes and Correlations

We coded 42 types of interactions between the forum participants. We hierarchized the codes into a model consisting of 15 categories. Our model differs in structure compared with other models in the literature. Although merging some codes into one category may seem unintuitive at first, it resulted from a modified combination of different theoretical approaches and multidisciplinary backgrounds of authors (psychiatry, psychology, sociology, epidemiology, and public health). For example, even though proanorexia forums supported harmful behaviors and contained reinforcers for further weight loss or praise for achieving lower weight, we decided to include the *supportive of harmful behaviors* category in the *behavior promotion* category. A membership in a social group or a web-based community affects the beliefs, preferences, and behaviors of the members via various mechanisms of social influence [163]. Therefore, the category of *behavior promotion* embraces all acts that reinforce behavior patterns regardless of their health consequences.

Assessing the co-occurrence of interactions, we found that the *request for information* most commonly co-occurred with *network*, which stems from the reciprocal nature of conversation that involves the exchange of questions and replies. The strongest associations were found for *requesting engagement* and *disagreement*, *relationship* and *confidentiality*, and *referring to the rules* and *rejection*. This may be explained by group processes that occur when new members join the group and are mobilized to share their story; the members are assured of confidentiality and presented with the rules that, for example, if violated, will result in the member being removed from the group [164].

A comparison of our codes with the terms identified in VOSviewer showed that our codebook may lack some interactions. However, these extra terms might have occurred in the background or discussion sections and do not apply to our study. Thus, VOSviewer clusters should always be interpreted together with content analysis.

Overall, our study showed that it is possible to use ML techniques to classify mental disorders based on secondary data. Although the results may seem satisfactory, as the accuracy for decision trees was >80%, we cannot consider them to be more than just a proof of concept because of several limitations.

### Our Results in the Context of Previous Research

To our knowledge, this is the first scoping review that comprehensively summarizes evidence on all types of web-based peer-to-peer interactions among people with mental disorders. Previous reviews addressed only some types of peer interactions in the context of various nonpsychiatric health-related conditions, such as spinal cord injury [165,166], breastfeeding of hospitalized infants [167], or cancer [168]. In secondary research, peer-to-peer interactions are mostly assessed quantitatively (eg, efficacy assessment [51-53]).

Our study was not limited to specific mental health problems. This is in contrast to previous reviews on mental conditions, as they mainly addressed suicide prevention and dementia. Bowersox et al [169] conducted a scoping review on the function of peers in the prevention of suicidal behaviors. The authors concluded that peer-based interventions could play an important role in suicide prevention. Schlichthorst et al [170] studied peer support programs in suicide prevention and emphasized the usefulness of internet forums as support for people with a history of suicide attempt. Moreover, they alerted to the risks of unmoderated websites.

Carter et al [171] and Newman et al [172] also reviewed web-based peer support interventions in the context of a specific mental health problem (ie, dementia). However, unlike our study, they did not focus on people who directly experienced these problems but assessed individuals who cared for people with dementia. In addition, they did not assess the quality of the included studies. Similar to our approach, they searched several databases and applied similar guidelines for reporting scoping studies [173]. However, they attempted to answer different questions about the effectiveness of interventions and their cost-effectiveness, in addition to identifying the gaps in knowledge.

### Limitations

Our study has several limitations. First, we believe that the use of ML techniques requires more data than those collected in this study. Nevertheless, we consider this analysis to be a proof of concept only. Second, the CASP tool was adapted to our needs by dividing the last question into 3 subquestions. This makes it more challenging to compare the quality of the included studies with that of similar studies. Moreover, with a small data set available, we used the same data for training and calculating the accuracy of ML algorithms (without external validation), which limits the reliability of the results.

### Strengths

The strengths of our study include the use of a broad question followed by comprehensive and rigorous search of eligible studies. We searched 4 databases and followed the reporting process provided by Tricco et al [54,55]. In addition, we also assessed the quality of all included studies. We proposed a codebook and partition tree based on the dispersion of the results and compared it with other models. This innovation helps standardize the evidence and allows for data comparison across studies. Finally, we applied ML techniques to identify mental disorders using interactions among peers. The results are quite satisfactory, and even though they are a proof of concept, they can be further explored in future studies.

### Future Research

We believe that our codebook describing the categories of peer-to-peer interactions defined in this study can be used in future in-depth investigations of individual mental disorders. In addition, by using AI techniques and applying the rigorous validation of accuracy, this type of analysis could be used to facilitate the diagnosis or screening of mental disorders within web-based self-help groups. Moreover, the assessment of the co-occurrence of interactions and types of disorders could help identify adequate skills and communication styles to define the moderator’s characteristics to meet the requirements of a particular forum. However, as this is a proof-of-concept investigation, more specific data are needed to achieve these goals. As only a few studies have investigated web-based peer-to-peer interactions in the setting of mental disorders, more primary research is needed to obtain more evidence. It would be helpful to develop an ML model to establish which interactions are associated with specific diseases and to use AI techniques to investigate more interactions. This could translate into creating a personalized health care experience for individuals with mental disorders.

### Conclusions

Internet forums offering peer-to-peer support in mental health attract a heterogeneous group of people. Interactions between the members are predominately positive. Although the use of the internet to seek support for health problems has become commonplace, scientific evidence on this phenomenon is scarce. In the future, AI-based analysis of interactions between the members of mental health forums and a better understanding of their needs could help moderators provide personalized support to internet users.

## Appendix

Multimedia Appendix 1 PRISMA-ScR (Preferred Reporting Items for Systematic Reviews and Meta-Analyses extension for Scoping Reviews) checklist.

Multimedia Appendix 2 Search strategies.

Multimedia Appendix 3 Other partition trees.

Multimedia Appendix 4 List of included studies.

Multimedia Appendix 5 List of ongoing studies.

Multimedia Appendix 6 List of excluded studies according to the reason for exclusion.

Multimedia Appendix 7 Characteristics of included studies.

Multimedia Appendix 8 Means of codes per category and SDs for analyzed models.

Multimedia Appendix 9 Heatmaps with codes.

Multimedia Appendix 10 Supplementary material.

Multimedia Appendix 11 Machine-learning results with confusion matrices.

Multimedia Appendix 12 Decision trees of prediction of diseases based on the presence of interaction.

Multimedia Appendix 13 Quality of included studies.

Multimedia Appendix 14 List of clusters and related terms from VOSviewer.

## Abbreviations

AI: artificial intelligence
CASP: Critical Appraisal Skills Programme
ML: machine learning
PRISMA: Preferred Reporting Items for Systematic Reviews and Meta-Analyses
PRISMA-ScR: Preferred Reporting Items for Systematic Reviews and Meta-Analyses extension for Scoping Reviews

## Data Availability

The code is available on GitHub [174]. The data supporting the findings of this study are available in Multimedia Appendices 1-14.
