# Supplementary material for: Meeting the Unmet Needs of Individuals With Mental Disorders: Scoping Review on Peer-to-Peer Web-Based Interactions
Source: JMIR Ment Health. 2022 Dec 5;9(12):e36056. doi: 10.2196/36056 (PMC9788841; doi:10.2196/36056)
Supplement: Multimedia Appendix 2 [file mental_v9i12e36056_app2.docx]

**This is a Multimedia Appendix to a full manuscript published in the JMIR Mental Health. For full copyright and citation information see** [**http://dx.doi.org/10.2196/36056**](http://dx.doi.org/10.2196/36056)

**Search strategies**

| **Medline Ovid** |
| --- |
| mental illness.mp  exp mental disorder  mental disorders.mp  psychiatric disorder.mp  1-4 /OR  peer support.mp  peer-support.mp  peer based.mp  peer-based.mp  peer led.mp  peer interaction*.mp  peer intervention*.mp  peer recovery support.mp  peer provider*.mp  service user run.mp  service user led.mp  service user involv*.mp  consumer case management.mp  assertive community treatment*.mp  budd*.mp  consumer-provider*.mp  peer.mp  peer-to-peer.mp  social network*.mp  virtual communit*.mp  support group*.mp  ((patients OR users) AND forum*).mp  ((online OR internet OR digital OR website OR Web-based OR computer OR eHealth) AND support).mp  6-28 /OR  online textual analysis.mp  (Qualitative AND (research* OR study OR method* OR analys*)).mp  Discourse analys*.mp OR DA.mp  Content analys*.mp  Qualitative social media research.mp  (Mixed-method social media AND (research OR analys*)).mp  Non-reactiv*.mp  correspondence analys*.mp  discursive approach*.mp  in-depth analys*.mp  text reduc*.mp  30-40 /OR  5 AND 29 AND 41 |
| **Embase** |
| ‘mental illness’:ti,ab  ‘mental disorder’/exp  ‘mental disorders’:ti,ab  ‘psychiatric disorder’:ti,ab  1-4 /OR  ‘peer support’:ti,ab  ‘peer-support’:ti,ab  ‘peer based’:ti,ab  ‘peer-based’:ti,ab  ‘peer led’:ti,ab  ‘peer interaction*’:ti,ab  ‘peer intervention*’:ti,ab  ‘peer recovery support’:ti,ab  ‘peer provider*’:ti,ab  ‘service user run’:ti,ab  ‘service user led’:ti,ab  ‘service user involv*’:ti,ab  ‘consumer case management’:ti,ab  ‘assertive community treatment*’:ti,ab  ‘budd*’:ti,ab  ‘consumer-provider*’:ti,ab  ‘peer’:ti,ab  ‘peer-to-peer’:ti,ab  ‘social network*’:ti,ab  ‘virtual communit*’:ti,ab  ‘support group*’:ti,ab  ((‘patients’ OR ‘users’) AND ‘forum*’):ti,ab  ((‘online’ OR ‘internet’ OR ‘digital’ OR ‘website’ OR ‘Web-based’ OR ‘computer’ OR ‘eHealth’) AND ‘support’):ti,ab  6-28 /OR  ‘online textual analysis’:ti,ab  (‘Qualitative’ AND (‘research*’ OR ‘study’ OR ‘method*’ OR ‘analys*’)):ti,ab  ‘Discourse analys*’:ti,ab OR ‘DA’:ti,ab  ‘Content analys*’:ti,ab  ‘Qualitative social media research‘:ti,ab  (‘Mixed-method social media’ AND (‘research’ OR ‘analys*’)):ti,ab  ‘Non-reactiv*’:ti,ab  ‘correspondence analys*’:ti,ab  ‘discursive approach*’:ti,ab  ‘in-depth analys*’:ti,ab  ‘text reduc*’:ti,ab  30-40 /OR  5 AND 29 AND 41 |
| **Cochrane** |
| #1 "peer support"  #2 peer-support  #3 "peer based"  #4 peer-based  #5 "peer led"  #6 "peer interaction"  #7 peer recovery support  #8 "peer provider"  #9 service user run  #10 service user led  #11 service user involv*  #12 consumer case management  #13 assertive community treatment*  #14 consumer-provider*  #15 peer-to-peer  #16 "social network"  #17 virtual communit*  #18 "support group*"  #19 "users forum"  #20 "patients forum"  #21 "eHealth support"  #22 "online support"  #23 "internet support"  #24 "digital support"  #25 "website support"  #26 "web-based support"  #27 "computer support"  #28 #1 OR #2 OR #3 OR #4 OR #5 OR #6 OR #7 OR #8 OR #9 OR #10 OR #11 OR #12 OR #13 #14 #15 OR #16 OR #17 OR #18 OR #19 OR #20 OR #21 OR #22 OR #23 OR #24 OR #25 OR #26 OR #27  #29 online textual analysis  #30 "Qualitative method"  #31 "qualitative research"  #32 "qualitative study"  #33 "qualitative analysis"  #34 "Discourse analysis"  #35 Content analysis  #36 Qualitative social media research  #37 (Mixed-method social media AND (research OR analys*))  #38 Non-reactiv*  #39 "correspondence analysis"  #40 discursive approach*  #41 in-depth analys*  #42 text reduction  #43 #29 OR #30 OR #31 OR #32 OR #33 OR #34 OR #35 OR #36 OR #37 OR #38 OR #39 OR #40 OR #41 OR #42  #44 "mental illness"  #45 "mental disorder"  #46 "psychiatric disorder"  #47 MeSH descriptor: [Mental Disorders] explode all trees  #48 #44 OR #45 OR # 46 OR #47  #49 #28 AND #43 AND #48 |
| **Web of Science** |
| TS=mental illness  TS= mental disorder  TS=psychiatric disorder  1-3 /OR  TS=peer support OR TS=peer-support OR TS=peer based OR TS=peer-based OR TS=peer led OR TS=peer interaction* OR TS=peer intervention* OR TS=peer recovery support OR TS=peer provider* OR TS=service user run OR TS=service user led OR TS=service user involv* OR TS=consumer case management OR TS=assertive community treatment* OR TS=budd* OR TS=consumer-provider* OR TS=peer OR TS=peer-to-peer OR TS=social network* OR TS=virtual communit* OR TS=support group* OR TS=((patients OR users) AND forum*) OR TS=((online OR internet OR digital OR website OR Web-based OR computer OR eHealth) AND support)  TS=online textual analysis OR TS=(Qualitative AND (research* OR study OR method* OR analys*)) OR TS=Discourse analys* OR TS=DA OR TS=Content analys* OR TS=Qualitative social media research OR TS=(Mixed-method social media AND (research OR analys*)) OR TS=Non-reactiv* OR TS=correspondence analys* OR TS=discursive approach* OR TS=in-depth analys* OR TS=text reduc*  4 AND 5 AND 6 |
