# Supplementary material for: Meeting the Unmet Needs of Individuals With Mental Disorders: Scoping Review on Peer-to-Peer Web-Based Interactions
Source: JMIR Ment Health. 2022 Dec 5;9(12):e36056. doi: 10.2196/36056 (PMC9788841; doi:10.2196/36056)
Supplement: Multimedia Appendix 3 [file mental_v9i12e36056_app3.docx]

**This is a Multimedia Appendix to a full manuscript published in the JMIR Mental Health. For full copyright and citation information see** [**http://dx.doi.org/10.2196/36056**](http://dx.doi.org/10.2196/36056)

Other partition trees

1. Model #1 (Cutrona)

|  | Category | Codes | | | | | | | | | | | | | | | | | | | |
| --- | --- | --- | --- | --- | --- | --- | --- | --- | --- | --- | --- | --- | --- | --- | --- | --- | --- | --- | --- | --- | --- |
| interactions | informational | request for information | verification the authenticity | encouraging disclosure | request for opinions / suggestions | request for other kinds of support | informational | practical tricks | warnings | referral | related to medication | related to professional help | related to side effects | illegal advices | referring to the rules | disagreement | situation appraisal | network | sharing self-­disclosure | clarifications | behaviors promotion |
|  | tangible | instrumental | tangible |  |  |  |  |  |  |  |  |  |  |  |  |  |  |  |  |  |  |
|  | esteem | compliment | encouragement / motivation |  |  |  |  |  |  |  |  |  |  |  |  |  |  |  |  |  |  |
|  | network | small talks / socializing | tension release / jokes | empathy / compassion | presence / companions | relationship | acceptance | requesting engagement | confidentiality |  |  |  |  |  |  |  |  |  |  |  |  |
|  | emotional | positive | negative | spiritual | reluctance / aversion | rejection | aggression | sarcastic comments | appreciation / gratitude | offering hope | apologizing |  |  |  |  |  |  |  |  |  |  |

1. Model #2 (Cutrona modified)

|  | Category | Subcategory | Codes | | | | | | | | | | | | | | |
| --- | --- | --- | --- | --- | --- | --- | --- | --- | --- | --- | --- | --- | --- | --- | --- | --- | --- |
| interactions | informational | requested | request for information | verification the authenticity | encouraging disclosure | request for opinions / suggestions | request for other kinds of support |  |  |  |  |  |  |  |  |  |  |
|  |  | offered | informational | practical tricks | warnings | referral | related to medication | related to professional help | related to side effects | illegal advices | referring to the rules | disagreement | situation appraisal | network | sharing self-­disclosure | clarifications | behaviors promotion |
|  | tangible | requested |  |  |  |  |  |  |  |  |  |  |  |  |  |  |  |
|  |  | offered | instrumental | tangible |  |  |  |  |  |  |  |  |  |  |  |  |  |
|  | esteem | requested |  |  |  |  |  |  |  |  |  |  |  |  |  |  |  |
|  |  | offered | compliment | encouragement / motivation | confidentiality |  |  |  |  |  |  |  |  |  |  |  |  |
|  | network | requested | requesting engagement |  |  |  |  |  |  |  |  |  |  |  |  |  |  |
|  |  | offered | small talks / socializing | tension release / jokes | empathy / compassion | presence / companions | relationship | acceptance |  |  |  |  |  |  |  |  |  |
|  | emotional | requested |  |  |  |  |  |  |  |  |  |  |  |  |  |  |  |
|  |  | offered | positive | negative | spiritual | reluctance / aversion | rejection | aggression | sarcastic comments | appreciation / gratitude | offering hope | apologizing |  |  |  |  |  |

1. Model #3 (Rime)

|  | Category | Subcategory | Codes | | | | | | | | | | | | |
| --- | --- | --- | --- | --- | --- | --- | --- | --- | --- | --- | --- | --- | --- | --- | --- |
| interactions | social exchange | seeking social contact | request for other kinds of support | apologizing |  |  |  |  |  |  |  |  |  |  |  |
|  |  | narration | sharing self-­disclosure | encouraging disclosure | requesting engagement | verification the authenticity |  |  |  |  |  |  |  |  |  |
|  |  | social Comparison | network | situation appraisal | small talks / socializing | tension release / jokes | empathy / compassion | presence / companions | acceptance |  |  |  |  |  |  |
|  | Attachment System Activation | | request for information | request for opinions / suggestions |  |  |  |  |  |  |  |  |  |  |  |
|  | emotional expression | | positive | negative | spiritual | reluctance / aversion | rejection | aggression | sarcastic comments | appreciation / gratitude | offering hope |  |  |  |  |
|  | contributing support and information | | informational | referring to the rules | referral | related to medication | related to professional help | related to side effects | instrumental | tangible | encouragement / motivation | practical tricks | warnings | illegal advices | confidentiality |
|  | contextual expression | | clarifications | disagreement | compliment | relationship | behaviors promotion |  |  |  |  |  |  |  |  |

1. Model #4 (Gaysynsky)

|  | Category | Codes | | | | | | | | | | | | | | | | | | | | | |
| --- | --- | --- | --- | --- | --- | --- | --- | --- | --- | --- | --- | --- | --- | --- | --- | --- | --- | --- | --- | --- | --- | --- | --- |
| interactions | expressions of gratitude | appreciation / gratitude |  |  |  |  |  |  |  |  |  |  |  |  |  |  |  |  |  |  |  |  |  |
|  | administrative / engagement | requesting engagement | encouragement / motivation |  |  |  |  |  |  |  |  |  |  |  |  |  |  |  |  |  |  |  |  |
|  | hanter | tension release / jokes |  |  |  |  |  |  |  |  |  |  |  |  |  |  |  |  |  |  |  |  |  |
|  | socializing | small talks / socializing | encouraging disclosure | request for other kinds of support |  |  |  |  |  |  |  |  |  |  |  |  |  |  |  |  |  |  |  |
|  | group cohesion | presence / companions | relationship | acceptance | network |  |  |  |  |  |  |  |  |  |  |  |  |  |  |  |  |  |  |
|  | negative interaction | reluctance / aversion | rejection | aggression | sarcastic comments | disagreement |  |  |  |  |  |  |  |  |  |  |  |  |  |  |  |  |  |
|  | community protection | empathy / compassion | confidentiality | referring to the rules | verification the authenticity |  |  |  |  |  |  |  |  |  |  |  |  |  |  |  |  |  |  |
|  | miscellaneous | offering hope | sharing self-­disclosure | compliment | behaviors promotion | apologizing | clarifications | situation appraisal | instrumental | tangible | informational | practical tricks | warnings | referral | related to medication | related to professional help | related to side effects | illegal advices | spiritual | request for information | request for opinions / suggestions | negative | positive |

1. Model #5 (adapted by Liu)

|  | Category | Subcategory | Codes | | | | | | | | | | | | | |
| --- | --- | --- | --- | --- | --- | --- | --- | --- | --- | --- | --- | --- | --- | --- | --- | --- |
| interactions | support | direct support request | request for other kinds of support | encouraging disclosure |  |  |  |  |  |  |  |  |  |  |  |  |
|  |  | emotional support | tension release / jokes | encouragement / motivation | offering hope | empathy / compassion | compliment | relationship | confidentiality | behaviors promotion | appreciation / gratitude | apologizing |  |  |  |  |
|  |  | informational support | clarifications | situation appraisal | network | referring to the rules | instrumental | tangible | informational | practical tricks | warnings | referral | related to medication | related to professional help | related to side effects | illegal advices |
|  |  | companionship support | presence / companions | acceptance | small talks / socializing | spiritual | requesting engagement |  |  |  |  |  |  |  |  |  |
|  |  | direct informational request | request for information | request for opinions / suggestions | verification the authenticity |  |  |  |  |  |  |  |  |  |  |  |
|  | disclosure | positive emotional disclosure | positive |  |  |  |  |  |  |  |  |  |  |  |  |  |
|  |  | negative emotional disclosure | negative | disagreement | reluctance / aversion | rejection | aggression | sarcastic comments |  |  |  |  |  |  |  |  |
|  |  | disclosure with recovery problem | sharing self-­disclosure |  |  |  |  |  |  |  |  |  |  |  |  |  |

1. Model #6 (Wang)

|  | Category | Subcategory | Codes | | | | | | | | | | | | | |
| --- | --- | --- | --- | --- | --- | --- | --- | --- | --- | --- | --- | --- | --- | --- | --- | --- |
| interactions | emotional self-disclosure | positive emotional self-disclosure | positive |  |  |  |  |  |  |  |  |  |  |  |  |  |
|  |  | negative emotional self-disclosure | negative |  |  |  |  |  |  |  |  |  |  |  |  |  |
|  | informational self-disclosure | positive informational self-disclosure | sharing self-­disclosure | appreciation / gratitude | apologizing |  |  |  |  |  |  |  |  |  |  |  |
|  |  | negative informational self-disclosure | disagreement | reluctance / aversion | rejection | aggression | sarcastic comments |  |  |  |  |  |  |  |  |  |
|  | asking a question | | request for other kinds of support |  |  |  |  |  |  |  |  |  |  |  |  |  |
|  | eliciting support | emotional support elicitation | requesting engagement |  |  |  |  |  |  |  |  |  |  |  |  |  |
|  |  | informational support elicitation | request for information | request for opinions / suggestions | encouraging disclosure | verification the authenticity |  |  |  |  |  |  |  |  |  |  |
|  | providing support | provide emotional support | tension release / jokes | small talks / socializing | encouragement / motivation | offering hope | empathy / compassion | presence / companions | compliment | relationship | confidentiality | behaviors promotion | acceptance | spiritual |  |  |
|  |  | provide informational support | clarifications | situation appraisal | network | referring to the rules | instrumental | tangible | informational | practical tricks | warnings | referral | related to medication | related to professional help | related to side effects | illegal advices |

1. Model #7 (Bales)

|  | Category | Code | | | | | | | | | | | |
| --- | --- | --- | --- | --- | --- | --- | --- | --- | --- | --- | --- | --- | --- |
| interactions | social emotional positive reactions | solidarity / seems friendly | spiritual | positive | offering hope | empathy / compassion | presence / companions | compliment | acceptance | encouragement / motivation | confidentiality | appreciation / gratitude |  |
|  |  | agree |  |  |  |  |  |  |  |  |  |  |  |
|  |  | tension release | tension release / jokes | small talks / socializing | apologizing |  |  |  |  |  |  |  |  |
|  | attempted answers | gives suggestions | practical tricks | warnings | illegal advices |  |  |  |  |  |  |  |  |
|  |  | gives opinions | behaviors promotion | relationship |  |  |  |  |  |  |  |  |  |
|  |  | gives orientation / information | instrumental | tangible | informational | referring to the rules | referral | related to medication | related to professional help | related to side effects | situation appraisal | network | sharing self-­disclosure |
|  | questions | ask for orientation / information | request for other kinds of support | request for information | clarifications | verification the authenticity | encouraging disclosure |  |  |  |  |  |  |
|  |  | ask for opinions | request for opinions / suggestions |  |  |  |  |  |  |  |  |  |  |
|  |  | ask for suggestions | requesting engagement |  |  |  |  |  |  |  |  |  |  |
|  | social emotional- negative reactions | disagrees | disagreement |  |  |  |  |  |  |  |  |  |  |
|  |  | show tension |  |  |  |  |  |  |  |  |  |  |  |
|  |  | show antagonism / seems unfriendly | reluctance / aversion | rejection | aggression | sarcastic comments | negative |  |  |  |  |  |  |

1. Model #8 (modified Greiner)

|  | Category | Subcategory | Codess | | | | | | | |
| --- | --- | --- | --- | --- | --- | --- | --- | --- | --- | --- |
| interactions | illness-related aspects | symptoms | related to side effects |  |  |  |  |  |  |  |
|  |  | medication and treatment | related to medication |  |  |  |  |  |  |  |
|  |  | professionals | related to professional help |  |  |  |  |  |  |  |
|  | social aspects | | relationship |  |  |  |  |  |  |  |
|  | financial and legal issues | financial issues | instrumental | tangible |  |  |  |  |  |  |
|  |  | housing | illegal advices |  |  |  |  |  |  |  |
|  | exchange of information | disclosure | encouraging disclosure | sharing self-­disclosure |  |  |  |  |  |  |
|  |  | provide information | practical tricks | warnings | behaviors promotion | informational | referral | referring to the rules |  |  |
|  |  | request information | request for other kinds of support | request for information | clarifications | verification the authenticity | request for opinions / suggestions | requesting engagement |  |  |
|  |  | gratitude | appreciation / gratitude |  |  |  |  |  |  |  |
|  | emotional support | | empathy / compassion | presence / companions | acceptance | offering hope | encouragement / motivation | spiritual | compliment | confidentiality |
|  | group support | online group cohesion | positive | network | situation appraisal | tension release / jokes | small talks / socializing | apologizing |  |  |
|  |  | negative statement | reluctance / aversion | rejection | aggression | sarcastic comments | negative | disagreement |  |  |

1. Model #9

|  | Category | Subcategory | Subsubcategory | Codes | | | | | | | | | |
| --- | --- | --- | --- | --- | --- | --- | --- | --- | --- | --- | --- | --- | --- |
| interactions | community dynamics | between | | sharing self-­disclosure | encouraging disclosure | requesting engagement | small talks / socializing | tension release / jokes | verification the authenticity | situation appraisal | network | apologizing | referring to the rules |
|  |  | type of reactions | positive | positive | appreciation / gratitude | encouragement / motivation | offering hope | empathy / compassion | presence / companions | compliment |  |  |  |
|  |  |  | negative | negative | disagreement | reluctance / aversion | rejection | aggression | sarcastic comments |  |  |  |  |
|  |  | values | | relationship | confidentiality | behaviors promotion | acceptance |  |  |  |  |  |  |
|  | support | request | | request for information | clarifications | request for opinions / suggestions | request for other kinds of support |  |  |  |  |  |  |
|  |  | offered | instrumental | instrumental | practical tricks | tangible |  |  |  |  |  |  |  |
|  |  |  | related to treatment | related to medication | related to professional help | related to side effects |  |  |  |  |  |  |  |
|  |  |  | informational | informational | warnings | referral | illegal advices |  |  |  |  |  |  |
|  |  |  | spiritual | spiritual |  |  |  |  |  |  |  |  |  |

1. Model #10

|  | Category | Subcategory | Subsubcategory | Codes | | | | | | | | | | | | |
| --- | --- | --- | --- | --- | --- | --- | --- | --- | --- | --- | --- | --- | --- | --- | --- | --- |
| interactions | offered | support | | instrumental | tangible | informational | practical tricks | warnings | referral | related to medication | related to professional help | related to side effects | illegal advices | spiritual |  |  |
|  |  | personal infos | | sharing self-­disclosure |  |  |  |  |  |  |  |  |  |  |  |  |
|  |  | reacting | positive | situation appraisal | network | appreciation / gratitude | apologizing | encouragement / motivation | offering hope | empathy / compassion | presence / companions | compliment | relationship | confidentiality | behaviors promotion | acceptance |
|  |  |  | negative | disagreement | reluctance / aversion | rejection | aggression | sarcastic comments |  |  |  |  |  |  |  |  |
|  | requested | support | | request for opinions / suggestions | request for other kinds of support | request for information | clarifications |  |  |  |  |  |  |  |  |  |
|  |  | personal infos | | requesting engagement | verification the authenticity | encouraging disclosure |  |  |  |  |  |  |  |  |  |  |
|  | other | emotional | | positive | negative |  |  |  |  |  |  |  |  |  |  |  |
|  |  | socializing | | small talks / socializing | tension release / jokes | referring to the rules |  |  |  |  |  |  |  |  |  |  |

1. Model #11

|  | Category | Subcategory | Codes | | | | | | | | | | | | | | | | | | | | | | | |
| --- | --- | --- | --- | --- | --- | --- | --- | --- | --- | --- | --- | --- | --- | --- | --- | --- | --- | --- | --- | --- | --- | --- | --- | --- | --- | --- |
| interactions | fact-based | requested | request for information | request for opinions / suggestions | verification the authenticity |  |  |  |  |  |  |  |  |  |  |  |  |  |  |  |  |  |  |  |  |  |
|  |  | offered | informational | referring to the rules | referral | related to medication | related to professional help | related to side effects | situation appraisal | network | clarifications |  |  |  |  |  |  |  |  |  |  |  |  |  |  |  |
|  | experience-based | requested | encouraging disclosure | request for other kinds of support |  |  |  |  |  |  |  |  |  |  |  |  |  |  |  |  |  |  |  |  |  |  |
|  |  | offered | practical tricks | warnings | illegal advices | sharing self-­disclosure |  |  |  |  |  |  |  |  |  |  |  |  |  |  |  |  |  |  |  |  |
|  | other | | instrumental | tangible | positive | negative | small talks / socializing | tension release / jokes | disagreement | behaviors promotion | compliment | encouragement / motivation | empathy / compassion | presence / companions | relationship | acceptance | requesting engagement | spiritual | reluctance / aversion | rejection | aggression | sarcastic comments | appreciation / gratitude | offering hope | confidentiality | apologizing |

1. Model #12

|  | Category | Codes | | | | | | | | | | | | | | | | | | | | | |
| --- | --- | --- | --- | --- | --- | --- | --- | --- | --- | --- | --- | --- | --- | --- | --- | --- | --- | --- | --- | --- | --- | --- | --- |
| interactions | positive | appreciation / gratitude | apologizing | encouragement / motivation | offering hope | empathy / compassion | presence / companions | compliment | relationship | confidentiality | behaviors promotion | acceptance | positive | spiritual |  |  |  |  |  |  |  |  |  |
|  | negative | disagreement | reluctance / aversion | rejection | aggression | sarcastic comments | negative | illegal advices |  |  |  |  |  |  |  |  |  |  |  |  |  |  |  |
|  | neutral | situation appraisal | network | request for opinions / suggestions | request for other kinds of support | request for information | clarifications | requesting engagement | verification the authenticity | encouraging disclosure | small talks / socializing | tension release / jokes | referring to the rules | instrumental | tangible | informational | practical tricks | warnings | referral | related to medication | related to professional help | related to side effects | sharing self-­disclosure |

1. Model #13

|  | Category | Codes | | | | | | | | | | | | | |
| --- | --- | --- | --- | --- | --- | --- | --- | --- | --- | --- | --- | --- | --- | --- | --- |
| interactions | support | instrumental | tangible | informational | practical tricks | warnings | referral | illegal advices | spiritual | referring to the rules |  |  |  |  |  |
|  | treatment related | related to medication | related to professional help | related to side effects |  |  |  |  |  |  |  |  |  |  |  |
|  | adverse responses / reactions | disagreement | reluctance / aversion | rejection | aggression | sarcastic comments |  |  |  |  |  |  |  |  |  |
|  | requests | request for opinions / suggestions | request for other kinds of support | request for information | clarifications | requesting engagement | verification the authenticity | encouraging disclosure |  |  |  |  |  |  |  |
|  | positive reactions | encouragement / motivation | situation appraisal | network | appreciation / gratitude | apologizing | offering hope | empathy / compassion | presence / companions | compliment | relationship | confidentiality | behaviors promotion | acceptance | small talks / socializing |
|  | disclosure | sharing self-­disclosure |  |  |  |  |  |  |  |  |  |  |  |  |  |
|  | emotions | positive | negative | tension release / jokes |  |  |  |  |  |  |  |  |  |  |  |
