# Supplementary material for: Meeting the Unmet Needs of Individuals With Mental Disorders: Scoping Review on Peer-to-Peer Web-Based Interactions
Source: JMIR Ment Health. 2022 Dec 5;9(12):e36056. doi: 10.2196/36056 (PMC9788841; doi:10.2196/36056)
Supplement: Multimedia Appendix 4 [file mental_v9i12e36056_app4.docx]

**This is a Multimedia Appendix to a full manuscript published in the JMIR Mental Health. For full copyright and citation information see** [**http://dx.doi.org/10.2196/36056**](http://dx.doi.org/10.2196/36056)

**List of included studies**

| 1. Andalibi, N., Ozturk, P., Forte, A., & Assoc Comp, M. (2017). Sensitive Self-disclosures, Responses, and Social Support on Instagram: The Case of #Depression. 2. Barney, L. J., Griffiths, K. M., & Banfield, M. A. (2011). Explicit and implicit information needs of people with depression: A qualitative investigation of problems reported on an online depression support forum. BMC psychiatry, 11. doi:10.1186/1471-244X-11-88 3. Beck, S. J., Paskewitz, E. A., Anderson, W. A., Bourdeaux, R., & Currie-Mueller, J. (2017). The Task and Relational Dimensions of Online Social Support. Health communication, 32(3), 347-355. doi:10.1080/10410236.2016.1138383 4. Bronstein, J. (2014). Is this OCD?: Exploring conditions of information poverty in online support groups dealing with obsessive compulsive disorder. Information Research-an International Electronic Journal, 19(4). 5. Brown, S.-E., & Altice, F. L. (2014). Self-management of buprenorphine/naloxone among online discussion board users. Substance use & misuse, 49(8), 1017-1024. doi:https://dx.doi.org/10.3109/10826084.2014.888449 6. Chen, A. T., Slattery, K., Tomasino, K. N., Rubanovich, C. K., Bardsley, L. R., & Mohr, D. C. (2020). Challenges and benefits of an internet-based intervention with a peer support component for older adults with depression: Qualitative analysis of textual data. Journal of medical internet research, 22(6). doi:10.2196/17586 7. Cunningham, J. A., van Mierlo, T., & Fournier, R. (2008). An online support group for problem drinkers: AlcoholHelpCenter.net. Patient education and counseling, 70(2), 193-198. 8. Doran, J., & Lewis, C. A. (2011). Posting incognito... males with eating problems: Online emotional expression and support. Journal of Cyber Therapy and Rehabilitation, 4(3), 341-350. 9. Edward, K.-L., & Robins, A. (2012). Dual diagnosis, as described by those who experience the disorder: using the Internet as a source of data. International journal of mental health nursing, 21(6), 550-559. doi:https://dx.doi.org/10.1111/j.1447-0349.2012.00833.x 10. Eghdam, A., Hamidi, U., Bartfai, A., & Koch, S. (2018). Facebook as communication support for persons with potential mild acquired cognitive impairment: A content and social network analysis study. Plos one, 13(1), e0191878. doi:https://dx.doi.org/10.1371/journal.pone.0191878 11. Evans, M., Donelle, L., & Hume-Loveland, L. (2012). Social support and online postpartum depression discussion groups: a content analysis. Patient education and counseling, 87(3), 405-410. doi:https://dx.doi.org/10.1016/j.pec.2011.09.011 12. Gajaria, A., Yeung, E., Goodale, T., & Charach, A. (2011). Beliefs about attention-deficit/hyperactivity disorder and response to stereotypes: youth postings in Facebook groups. The Journal of adolescent health : official publication of the Society for Adolescent Medicine, 49(1), 15-20. doi:https://dx.doi.org/10.1016/j.jadohealth.2010.09.004 13. Gavin, J., Rodham, K., & Poyer, H. (2008). The presentation of "pro-anorexia" in online group interactions. Qualitative health research, 18(3), 325-333. doi:https://dx.doi.org/10.1177/1049732307311640 14. Giles, D. C., & Newbold, J. (2011). Self- and other-diagnosis in user-led mental health online communities. Qualitative health research, 21(3), 419-428. doi:https://dx.doi.org/10.1177/1049732310381388 15. Greiner, C., Chatton, A., & Khazaal, Y. (2017). Online self-help forums on cannabis: A content assessment. Patient education and counseling, 100(10), 1943-1950. doi:10.1016/j.pec.2017.06.001 16. Horgan, A., McCarthy, G., & Sweeney, J. (2013). An Evaluation of an Online Peer Support Forum for University Students With Depressive Symptoms. Archives of psychiatric nursing, 27(2), 84-89. doi:10.1016/j.apnu.2012.12.005 17. Juarascio, A. S., Shoaib, A., & Timko, C. A. (2010). Pro-eating disorder communities on social networking sites: a content analysis. Eating disorders, 18(5), 393-407. doi:https://dx.doi.org/10.1080/10640266.2010.511918 18. Kantrowitz-Gordon, I. (2013). Internet confessions of postpartum depression. Issues in mental health nursing, 34(12), 874-882. doi:https://dx.doi.org/10.3109/01612840.2013.806618 19. Kendal, S., Kirk, S., Elvey, R., Catchpole, R., & Pryjmachuk, S. (2017). How a moderated online discussion forum facilitates support for young people with eating disorders. Health expectations : an international journal of public participation in health care and health policy, 20(1), 98-111. doi:https://dx.doi.org/10.1111/hex.12439 20. Keski-Rahkonen, A., & Tozzi, F. (2005). The process of recovery in eating disorder sufferers' own words: an Internet-based study. The International journal of eating disorders, 37 Suppl, S80-89. 21. Lavis, A., & Winter, R. (2020). #Online harms or benefits? An ethnographic analysis of the positives and negatives of peer-support around self-harm on social media. Journal of Child Psychology and Psychiatry, 61(8), 842-854. doi:10.1111/jcpp.13245 22. Lerman, B. I., Lewis, S. P., Lumley, M., Grogan, G. J., Hudson, C. C., & Johnson, E. (2017). Teen Depression Groups on Facebook: A Content Analysis. Journal of Adolescent Research, 32(6), 719-741. doi:10.1177/0743558416673717 23. Liu, Y., Kornfield, R., Shaw, B. R., Shah, D. V., McTavish, F., & Gustafson, D. H. (2017). When support is needed: Social support solicitation and provision in an online alcohol use disorder forum. Digital health, 3. doi:10.1177/2055207617704274 24. Lord, V. M., Reiboldt, W., Gonitzke, D., Parker, E., & Peterson, C. (2018). Experiences of recovery in binge-eating disorder: a qualitative approach using online message boards. Eating and weight disorders : EWD, 23(1), 95-105. doi:https://dx.doi.org/10.1007/s40519-016-0335-z 25. Moore, D., Ayers, S., & Drey, N. (2016). A Thematic Analysis of Stigma and Disclosure for Perinatal Depression on an Online Forum. JMIR mental health, 3(2). doi:10.2196/mental.5611 26. Mudry, T. E., & Strong, T. (2013). Doing recovery online. Qualitative health research, 23(3), 313-325. doi:https://dx.doi.org/10.1177/1049732312468296 27. Naslund, J. A., Grande, S. W., Aschbrenner, K. A., & Elwyn, G. (2014). Naturally occurring peer support through social media: the experiences of individuals with severe mental illness using YouTube. Plos one, 9(10), e110171. doi:https://dx.doi.org/10.1371/journal.pone.0110171 28. Park, A., Conway, M., & Chen, A. T. (2018). Examining thematic similarity, difference, and membership in three online mental health communities from reddit: A text mining and visualization approach. Computers in Human Behavior, 78, 98-112. doi:10.1016/j.chb.2017.09.001 29. Prescott, J., Rathbone, A. L., & Brown, G. (2020). Online peer to peer support: Qualitative analysis of UK and US open mental health Facebook groups. Digital health, 6. doi:10.1177/2055207620979209 30. Salzmann-Erikson, M., & Hicdurmaz, D. (2017). Use of Social Media Among Individuals Who Suffer From Post-Traumatic Stress: A Qualitative Analysis of Narratives. Qualitative health research, 27(2), 285-294. 31. Sharkey, S., Smithson, J., Hewis, E., Jones, R., Emmens, T., Ford, T., & Owens, C. (2012). Supportive interchanges and face-work as 'protective talk' in an online self-harm support forum. Communication & medicine, 9(1), 71-82. 32. Smithson, J., Sharkey, S., Hewis, E., Jones, R. B., Emmens, T., Ford, T., & Owens, C. (2011). Membership and boundary maintenance on an online self-harm forum. Qualitative health research, 21(11), 1567-1575. 33. Soussan, C., & Kjellgren, A. (2014). Harm reduction and knowledge exchange-a qualitative analysis of drug-related Internet discussion forums. Harm reduction journal, 11, 25. doi:https://dx.doi.org/10.1186/1477-7517-11-25 34. Sowles, S. J., McLeary, M., Optican, A., Cahn, E., Krauss, M. J., Fitzsimmons-Craft, E. E., . . . Cavazos-Rehg, P. A. (2018). A content analysis of an online pro-eating disorder community on Reddit. Body image, 24, 137-144. doi:https://dx.doi.org/10.1016/j.bodyim.2018.01.001 35. Struik, L. L., & Baskerville, N. B. (2014). The role of Facebook in Crush the Crave, a mobile- and social media-based smoking cessation intervention: qualitative framework analysis of posts. Journal of medical internet research, 16(7), e170. doi:https://dx.doi.org/10.2196/jmir.3189 36. Walstrom, M. K. (2000). 'You know, who's the thinnest?': Combating surveillance and creating safety in coping with eating disorders online. Cyberpsychology and Behavior, 3(5), 761-783. doi:10.1089/10949310050191755 37. Worley, J., & Krishnan, D. (2020). Fighting the Battle of Recovery Together: A Content Analysis of Anonymous Posts in an Online Substance Use Forum. Issues in mental health nursing, 41(2), 102-112. doi:10.1080/01612840.2019.1646364 38. Yip, J. W. C. (2019). Evaluating the Communication of Online Social Support: A Mixed-Methods Analysis of Structure and Content. Health communication, 1-9. doi:https://dx.doi.org/10.1080/10410236.2019.1623643 39. Elran-Barak, R. (2021). Analyses of posts written in online eating disorder and depression/anxiety moderated communities: Emotional and informational communication before and during the COVID-19 outbreak. Internet Interventions, 26, 100438. 40. Flickinger, T. E., Waselewski, M., Tabackman, A., Huynh, J., Hodges, J., Otero, K., … & Dillingham, R. (2022). Communication between patients, peers, and care providers through a mobile health intervention supporting medication-assisted treatment for opioid use disorder. Patient Education and Counseling. 41. Goh, A. Q. Y., Lo, N. Y. W., Davis, C., & Chew, E. C. S. (2022). # EatingDisorderRecovery: a qualitative content analysis of eating disorder recovery-related posts on Instagram. Eating and Weight Disorders-Studies on Anorexia, Bulimia and Obesity, 27(4), 1535-1545. 42. Kim, H. S., Chung, M. Y., Rhee, E. S., & Kim, Y. (2022). Is it reciprocating or self-serving?: Understanding coping strategies for postpartum depression in an online community for Korean mothers. Health Care for Women International, 1-18. 43. Albano, G., Bonfanti, R. C., Gullo, S., Salerno, L., & Coco, G. L. (2021). The psychological impact of COVID-19 on people suffering from dysfunctional eating behaviours: a linguistic analysis of the contents shared in an online community during the lockdown. Research in Psychotherapy: Psychopathology, Process, and Outcome, 24(3). 44. Sik, D., Németh, R., & Katona, E. (2021). Topic modelling online depression forums: beyond narratives of self-objectification and self-blaming. Journal of Mental Health, 1-10. |
| --- |
