# Supplementary material for: Meeting the Unmet Needs of Individuals With Mental Disorders: Scoping Review on Peer-to-Peer Web-Based Interactions
Source: JMIR Ment Health. 2022 Dec 5;9(12):e36056. doi: 10.2196/36056 (PMC9788841; doi:10.2196/36056)
Supplement: Multimedia Appendix 5 [file mental_v9i12e36056_app5.docx]

**This is a Multimedia Appendix to a full manuscript published in the JMIR Mental Health. For full copyright and citation information see** [**http://dx.doi.org/10.2196/36056**](http://dx.doi.org/10.2196/36056)

**List of ongoing studies**

| 1. Bendall, S., Truss, K., Liao, J., & Phillips, L. (2018). What stops young people from seeking help for the effects of trauma? A qualitative analysis of internet forums. Early intervention in psychiatry, 12, 84. doi:10.1111/eip.12723 2. McGregor, K. A., & Clancy, O. (2019). Starving For Support: Natural Language Processing And Machine Learning Analysis of Anorexia Nervosa In Pro-Eating Disorder Communities. Journal of adolescent health, 64(2), S53. doi:10.1016/j.jadohealth.2018.10.116 3. Parsons, C., & McGrath, D. (2019). Medication information needs of people with Parkinson's disease and their carers: A qualitative study using an online discussion forum. International Journal of Pharmacy Practice, 27, 9. doi:10.1111/ijpp.12532 4. Žaja, N., Vukušić Rukavina, T., Brborović, O., Uzun, S., & Mimica, N. (2018). Reasons for using depression internet forums in Croatia. European psychiatry, 48, S416-S417. doi:10.1016/j.eurpsy.2017.12.023 5. Zhu, L., Mehta, N., Wu, W., Savage, R., Lam, K., Ghuman, I., . . . Rochon, P. A. (2019). DEMENTIA 2.0: AN EXPLORATORY ANALYSIS OF TWITTER AND HEALTH FORUM DATA. Alzheimer's and Dementia, 15(7), P1456. doi:10.1016/j.jalz.2019.06.4073 6. Dixon‐Ward, K. C., & Chan, S. W. (2022). Faking it’: Exploring adolescent perceptions of depression (in) authenticity and ‘attention seeking. British journal of clinical psychology, 61(2), 177-196. 7. Kepner, W., Meacham, M. C., & Nobles, A. L. (2022). Types and Sources of Stigma on Opioid Use Treatment and Recovery Communities on Reddit. Substance Use & Misuse, 57(10), 1511-1522. 8. Bickerstaff, J. M., Karim, S., Whitman, R. K., Cypher, A., Wiener, S., & Radovic, A. (2021). “You Have People Here to Help You, People Like Me”: a Qualitative Analysis of a Blogging Intervention for Adolescents and Young Adults with Depression or Anxiety. Journal of technology in behavioral science, 6(4), 578-588. |
| --- |
