# Supplementary material for: Meeting the Unmet Needs of Individuals With Mental Disorders: Scoping Review on Peer-to-Peer Web-Based Interactions
Source: JMIR Ment Health. 2022 Dec 5;9(12):e36056. doi: 10.2196/36056 (PMC9788841; doi:10.2196/36056)
Supplement: Multimedia Appendix 6 [file mental_v9i12e36056_app6.docx]

**This is a Multimedia Appendix to a full manuscript published in the JMIR Mental Health. For full copyright and citation information see** [**http://dx.doi.org/10.2196/36056**](http://dx.doi.org/10.2196/36056)

**List of excluded studies according to reason for exclusion**

| Irrelevant study design (n=16) |
| --- |
| 1. Abel, S., Machin, T., & Brownlow, C. (2019). Support, socialise and advocate: An exploration of the stated purposes of Facebook autism groups. Research in Autism Spectrum Disorders, 61, 10-21. doi:10.1016/j.rasd.2019.01.009 2. Athanasopoulou, C., & Sakellari, E. (2015). Facebook and Health Information: Content Analysis of Groups Related to Schizophrenia. Studies in health technology and informatics, 213, 255-258. 3. Fraga, B. S., da Silva, A. P. C., Murai, F., & Ieee. (2018). Online Social Networks in Health Care: A Study of Mental Disorders on Reddit. 4. Lamy, F. R., Daniulaityte, R., Nahhas, R. W., Barratt, M. J., Smith, A. G., Sheth, A., . . . Carlson, R. G. (2017). Increased in synthetic cannabinoids-related harms: Results from a longitudinal web-based content analysis. International Journal of Drug Policy, 44, 121-129. doi:10.1016/j.drugpo.2017.05.007 5. Linden, M., & Pirsich, C. (2017). Negative and helpful statements in a patient only or therapist guided internet forum in the aftercare for psychosomatic inpatients. Psychiatria Danubina, 29(4), 446-450. doi:10.24869/psyd.2017.446 6. Lyons, M., Aksayli, N. D., & Brewer, G. (2018). Mental distress and language use: Linguistic analysis of discussion forum posts. Computers in Human Behavior, 87, 207-211. doi:10.1016/j.chb.2018.05.035 7. Martinez-Perez, B., de la Torre-Diez, I., Bargiela-Florez, B., Lopez-Coronado, M., & Rodrigues, J. (2015). Content analysis of neurodegenerative and mental diseases social groups. Health informatics journal, 21(4), 267-283. doi:10.1177/1460458214525615 8. Mazel, S., Zisman-Ilani, Y., Hennig, S., Garnick, D., & Nicholson, J. (2021). Virtual Engagement in a Social Media Community of Mothers With Substance Use Disorders: Content Analysis. JMIR formative research, 5(6), e24353. doi:https://dx.doi.org/10.2196/24353 9. McCaig, D., Elliott, M. T., Siew, C. S. Q., Walasek, L., & Meyer, C. (2019). Profiling Commenters on Mental Health-Related Online Forums: A Methodological Example Focusing on Eating Disorder-Related Commenters. JMIR mental health, 6(4). doi:10.2196/12555 10. Moessner, M., Feldhege, J., Wolf, M., & Bauer, S. (2018). Analyzing big data in social media: Text and network analyses of an eating disorder forum. The International journal of eating disorders, 51(7), 656-667. doi:https://dx.doi.org/10.1002/eat.22878 11. Mokkenstorm, J. K., Mérelle, S. Y. M., Smit, J. H., Beekman, A. T. F., Kerkhof, A. J. F. M., Huisman, A., & Gilissen, R. (2019). Exploration of Benefits and Potential Harmful Effects of an Online Forum for Visitors to the Suicide Prevention Platform in The Netherlands. Crisis, 1-9. doi:10.1027/0227-5910/a000627 12. Nguyen, T., O'Dea, B., Larsen, M., Phung, D., Venkatesh, S., & Christensen, H. (2017). Using linguistic and topic analysis to classify sub-groups of online depression communities. Multimedia Tools and Applications, 76(8), 10653-10676. doi:10.1007/s11042-015-3128-x 13. Selby, P., van Mierlo, T., Voci, S. C., Parent, D., & Cunningham, J. A. (2010). Online social and professional support for smokers trying to quit: an exploration of first time posts from 2562 members. Journal of medical internet research, 12(3), e34. doi:https://dx.doi.org/10.2196/jmir.1340 14. Tan, Y. T., Rehm, I. C., Stevenson, J. L., & De Foe, A. (2021). Social Media Peer Support Groups for Obsessive-Compulsive and Related Disorders: Understanding the Predictors of Negative Experiences. Journal of affective disorders, 281, 661-672. doi:10.1016/j.jad.2020.11.094 15. Winkel, S., Groen, G., & Petermann, F. (2005). Social support in suicide forums. Praxis der Kinderpsychologie und Kinderpsychiatrie, 54(9), 714-727. 16. Xu, R. H., & Zhang, Q. P. (2016). Understanding Online Health Groups for Depression: Social Network and Linguistic Perspectives. Journal of medical internet research, 18(3). doi:10.2196/jmir.5042   Irrelevant intervention (n=12) |
| 1. Ahlstrom, B. H., & Wentz, E. (2014). Difficulties in everyday life: young persons with attention-deficit/hyperactivity disorder and autism spectrum disorders perspectives. A chat-log analysis. International journal of qualitative studies on health and well-being, 9, 23376. doi:https://dx.doi.org/10.3402/qhw.v9.23376 2. Carron-Arthur, B., Reynolds, J., Bennett, K., Bennett, A., & Griffiths, K. M. (2016). What's all the talk about? Topic modelling in a mental health Internet support group. BMC psychiatry, 16(1), 367. 3. Cavazos-Rehg, P. A., Krauss, M. J., Sowles, S., Connolly, S., Rosas, C., Bharadwaj, M., & Bierut, L. J. (2016). A content analysis of depression-related tweets. Computers in Human Behavior, 54, 351-357. doi:10.1016/j.chb.2015.08.023 4. Cohn, A. M., Amato, M. S., Zhao, K., Wang, X., Cha, S., Pearson, J. L., . . . Graham, A. L. (2019). Discussions of Alcohol Use in an Online Social Network for Smoking Cessation: Analysis of Topics, Sentiment, and Social Network Centrality. Alcoholism-Clinical and Experimental Research, 43(1), 108-114. doi:10.1111/acer.13906 5. Davis, S., & Lewis, C. A. (2019). Addiction to Self-harm? The Case of Online Postings on Self-harm Message Boards. International Journal of Mental Health and Addiction, 17(4), 1020-1035. doi:10.1007/s11469-018-9975-8 6. Dempsey, M., Foley, S., Frost, N., Murphy, R., Willis, N., Robinson, S., . . . McCarthy, J. Am I lazy, a drama queen or depressed? A journey through a pluralistic approach to analysing accounts of depression. Qualitative Research in Psychology. doi:10.1080/14780887.2019.1677833 7. Feuston, J. L., & Assoc Comp, M. (2019). Algorithms, Oppression, and Mental Illness on Social Media. 8. Mancera, K. C., De Santacruz, C., & Salamanca, M. A. (2014). ["Accepting Demented Minds". Opinion Group, Information and Support on Stigma of Mental Illness on Facebook]. <>. Grupo de opinion, informacion y apoyo sobre el estigma de las enfermedades mentales en Facebook., 43(3), 139-145. doi:https://dx.doi.org/10.1016/j.rcp.2014.02.007 9. Mitchell, J. T., Sweitzer, M. M., Tunno, A. M., Kollins, S. H., & McClernon, F. J. (2016). "I Use Weed for My ADHD": A Qualitative Analysis of Online Forum Discussions on Cannabis Use and ADHD. Plos one, 11(5), e0156614. doi:https://dx.doi.org/10.1371/journal.pone.0156614 10. Schmidt, M., Dillon, P. J., Jackson, B. M., Pirkey, P., & Kedia, S. K. (2019). "Gave me a line of ice and I got hooked": Exploring narratives of initiating methamphetamine use. Public health nursing (Boston, Mass.), 36(1), 18-27. doi:https://dx.doi.org/10.1111/phn.12568 11. Tran, T. B., Uebelacker, L., Wenze, S. J., Collins, C., & Broughton, M. K. (2015). Adaptive and Maladaptive Means of Using Facebook: A Qualitative Pilot Study to Inform Suggestions for Development of a Future Intervention for Depression. Journal of psychiatric practice, 21(6), 458-473. doi:https://dx.doi.org/10.1097/PRA.0000000000000109 12. Valeriani, G., Corazza, O., Bersani, F. S., Melcore, C., Metastasio, A., Bersani, G., & Schifano, F. (2015). Olanzapine as the ideal "trip terminator"? Analysis of online reports relating to antipsychotics' use and misuse following occurrence of novel psychoactive substance-related psychotic symptoms. Human psychopharmacology, 30(4), 249-254. doi:<https://dx.doi.org/10.1002/hup.2431> 13. Sedgewick, F., Leppanen, J., Austin, A., & Tchanturia, K. (2021). Different pathways, same goals: A large‐scale qualitative study of autistic and non‐autistic patient‐generated definitions of recovery from an eating disorder. European Eating Disorders Review. |
| Irrelevant population (n=4) |
| 1. Coulson, N. S., Buchanan, H., & Aubeeluck, A. (2007). Social support in cyberspace: a content analysis of communication within a Huntington's disease online support group. Patient education and counseling, 68(2), 173-178. 2. Hucker, A., & McCabe, M. P. (2014). A qualitative evaluation of online chat groups for women completing a psychological intervention for female sexual dysfunction. Journal of sex & marital therapy, 40(1), 58-68. doi:https://dx.doi.org/10.1080/0092623X.2012.675020 3. Li, A., Jiao, D., Liu, X., & Zhu, T. (2020). A Comparison of the Psycholinguistic Styles of Schizophrenia-Related Stigma and Depression-Related Stigma on Social Media: Content Analysis. Journal of medical internet research, 22(4), e16470. doi:https://dx.doi.org/10.2196/16470 4. Mbao, M., Collins-Pisano, C., & Fortuna, K. (2021). Older Adult Peer Support Specialists' Age-Related Contributions to an Integrated Medical and Psychiatric Self-Management Intervention: Qualitative Study of Text Message Exchanges. JMIR formative research, 5(3), e22950. doi:https://dx.doi.org/10.2196/22950 5. Evans, K., Rennick-Egglestone, S., Cox, S., Kuipers, Y., & Spiby, H. (2022). Remotely delivered interventions to support women with symptoms of anxiety in pregnancy: mixed methods systematic review and meta-analysis. Journal of medical Internet research, 24(2), e28093. |
