# Supplementary material for: Meeting the Unmet Needs of Individuals With Mental Disorders: Scoping Review on Peer-to-Peer Web-Based Interactions
Source: JMIR Ment Health. 2022 Dec 5;9(12):e36056. doi: 10.2196/36056 (PMC9788841; doi:10.2196/36056)
Supplement: Multimedia Appendix 7 [file mental_v9i12e36056_app7.docx]

**This is a Multimedia Appendix to a full manuscript published in the JMIR Mental Health. For full copyright and citation information see** [**http://dx.doi.org/10.2196/36056**](http://dx.doi.org/10.2196/36056)

Characteristics of included studies

| Study ID | Country of corresponding author | Study design | Place of interaction  1=forum  2=chat  3=media  4=support group  5=other | Who is the medium designed for? | Age  mean (range) | % of females | Type of analysis used | Coding schemes for interaction or social support | Method of coding  1=inductive  2=deductive  3=mixed | Number of users | Number of analyzed topics (’threads’) | Number of analyzed comments (’posts’) | CASP score | Conflict of interest | Funding |
| --- | --- | --- | --- | --- | --- | --- | --- | --- | --- | --- | --- | --- | --- | --- | --- |
| Albano 2021 | Italy | Q with FA | 4, MoodTech | ED | NR | 86 | 1; 10, linguistic | NR | NR | 23 | NR | NR | 2 | Reported | Government |
| Andalibi 2017 | United States | Q with FA | 3, Instagram | depression | NR | NR | 5, visual content analysis; 5, caption content analysis; 10, relation to image | Cutrona and Suhr | 1, iterative | 24920 | 788 | 1741 | 3 | NR | Government |
| Barney 2011 | Australia | Q with FA | 1, blueboard.anu.edu.au | depression | NR | NR | 1 | NR | 1 | 134 | NR | 2680 | 3 | None | Government |
| Beck 2016 | United States | mixed | 4, DailyStrength.org | depression | NR | NR | 5 | NR | NR | NR | 101 | 9226 | 3 | NR | NR |
| Bronstein 2014 | Israel | Q with FA | 4, Psychforums; 4, Daily Strength | OCD | NR | NR | 5, direct | NR | 2 | NR | NR | 202 | 1 | NR | NR |
| Brown 2014 | United States | Q | 1, multiple* | opioid addiction | NR | NR | 5, content with grounded theory | NR | 3, axial | NR | NR | NR | 3 | None | Government, Institutional |
| Chen 2020 | United States | Q with FA | 4, MoodTech | depression | 69.5 (SD 4.3) | 69.6 | 8; 10, grounded | NR | 1 | 23 | NR | NR | 2 | Reported | Governement |
| Cunningham 2007 | Canada | Q with FA | 1, AlcoholHelpCenter.net | alcohol addiction | NR | NR | NR | 5, Morse & Field, 1995 | NR | 155 | NR | 474 | 3 | Reported | NR |
| Doran 2011 | United Kingdom | Q | 1, Message board | ED | NR | 0 | 5 | NR | 1 | 15 | 19 | 101 | 1 | NR | NR |
| Edward 2012 | Australia | Q | 1, two online forums dedicated to peer support groups for dual diagnosis | dual diagnosis | NR | NR | 5 | Cutrona and Suhr; own coding scheme for interaction, Gaysynsky et al. | 1 | 60 | NR | 108 | 2 | NR | NR |
| Eghdam 2018 | Sweden | Q with FA | 3, facebook | MCI | NR | NR | 5, direct; 10, social network analysis | 3, Oakley | NR | 1310 | 630 | 4323 | 2 | None | Institutional |
| Elran-Barak 2021 | Israel | mixed | 1, Camoni | Depression, anxietydisorders, ED | NR | NR | 5 | NR | 3 | NR | NR | 1360 | 1 | None | Government |
| Evans 2012 | Canada | Q with FA | 4, NR | PPD | NR | NR | 5, directed qualitative content analysis | NR | NR | NR | NR | 512 | 2 | NR | NR |
| Flickinger 2022 | United States | Q with FA | 2, community message board | OUD | 33.7 | 48 | 5 | NR | 1 | 25 | NR | 49 | 1 | Reported | Government |
| Gajaria 2011 | Canada | Q with FA | 3, Facebook ADHD groups | ADHD | NR | NR | 5, ethnographic content analysis | NR | 1 | 2500 | NR | 479 | 2 | NR | Government |
| Gavin 2008 | United Kingdom | Q | 1, NR | anorexia | NR | 100 | 8 | NR | NR | 70 | NR | NR | 2 | NR | NR |
| Giles 2011 | United Kingdom | Q | 1, sites with forum or board, e.g. www.facetheissue.com, www.addicted.com | health issues and life challenges | NR | NR | 10, rhetorical analysis | 3, Bauer ar al.., Haker et al., Finn and Perron | NR | NR | NR | NR | 1 | None | Institutional |
| Goh 2022 | Singapore | Q with FA | 3, Instagram | Anorexia, bulimia, BED | 19 | 97.2 | 1; 5 | NR | 1 | 360 | 5 | 405 | 2 | None | None |
| Greiner 2017 | Switzerland | mixed | 1, multiple$ | cannabis addiction | 31 (18-78) | 24 | 5, content assessment | NR | 2 | 328 | NR | 717 | 3 | None | None |
| Horgan 2013 | Ireland | mixed | 1, www.losetheblues.ie | depression | 20.6 (18-24) | 35.6 | 1 | NR | NR, no info about any codes | 118 | NR | 53 | 3 | NR | NR |
| Juarascio 2010 | United States | Q | 3, Facebook, MySpace | anorexia | NR | NR | 5 | NR | 1 | NR | NR | NR | 2 | NR | NR |
| Kantrowitz-Gordon 2013 | United States | Q | 1, www.experienceproject.com | PPD | NR (16-55) | 97.06 | 7 | NR | NR | NR | NR | 102 | 3 | None | NR |
| Kendal 2016 | United Kingdom | Q | 1 | ED | NR | NR | 1 | NR | 1 | 119 | 97 | 420 | 2 | NR | Government |
| Keski-Rahkonen 2005 | United States | Q with FA | 1, a Finnish-language eating disorders discussion group | ED | median 21 (13-53) | 98,1 | 3, constant comparative method | NR | 1 | 158 | NR | 685 | 2 | NR | Institutional |
| Kim 2022 | United States | mixed | 1, Momsholic Baby | PPD | NR | NR | 5 | Cutrona and Suhr | 3 | NR | NR | 1676 | 2 | NR | None |
| Lavis 2020 | United Kingdom | Q | 3, Twitter, Reddit and Instagram | self-harm | NR | NR | 1 | NR | NR | NR | 10169 | 36934 | 1 | None | Not-for-profit foundation |
| Lerman 2016 | Canada | Q with FA | 3, six active Facebook groups for teen depression | depression | NR | NR | 5 | 3, Cohen and Wills | 3 | NR | NR | 508 | 2 | None | None |
| Liu 2017 | United States | mixed | 1, online alcohol use disorder forum | alcohol addiction | 38 (20-64) | 44 | 5 | NR | 2 | 170 | NR | 2590 | 2 | None | Government |
| Lord 2016 | United States | Q | 1, online support forum for ED recovery | BED | NR | NR | 1 | NR | 1 | 65 | NR | 681 | 3 | None | NR |
| Moore 2016 | United Kingdom | Q with FA | 1, Mumsnet | PPD | NR | NR | 1, deductive systematic thematic analysis from a realist stance | NR | 2 | NR | 102 | 1546 | 3 | None | NR |
| Mudry 2012 | Canada | Q | 1, NR | GA | NR | NR | 7; 10, ethnomethodology; 10, netnography | NR | NR | 11 | NR | 1791 | 2 | None | Institutional |
| Naslund 2014 | United States | Q | 3, YouTube | bipolar disorder; schizophrenia; schizoaffective disorder | NR (18-55) | NR | 1 | NR | 1 | NR | 19 | 3044 | 2 | None | None |
| Park 2017 | United States | Q with FA | 1, three Reddit communities (r/Anxiety, r/Depression, and r/PTSD). | depression; anxiety disorders; PTSD | NR | NR | 1 | NR | 1 | 41967 | 7410 | 132599 | 2 | NR | NR |
| Prescott 2020 | United Kingdom | Q with FA | 3, Facebook | depression; bipolar disorder; anxiety;  substance abuse  general mental health | NR | NR | 1; 5, textual | NR | 1 | NR | NR | NR | 2 | None | Institutional |
| Salzmann-Erikson 2016 | Sweden | Q | 1; 5, blog; 5, YouTube | PTSD | NR | NR | 5, netnographic method LiLEDDA | NR | 2 | NR | 29 | NR | 3 | None | None |
| Sharkey 2012 | United Kingdom | Q | 1, SharpTalk | self-harm | NR (16-25) | NR | 7 | 0 | NR | 77 | NR | NR | 2 | NR | Government |
| Sik 2021 | Hungary | mixed | 1, several& | depression | NR | NR | 5; 10, deep reading | NR | 1, LDA | NR | NR | NR | 1 | None | Government |
| Smithson 2011 | United Kingdom | Q | 1, SharpTalk | self-harm | NR | NR | 6; 7; 10, membership categorization analysis | NR | NR | 77 | NR | NR | 2 | None | Government |
| Soussan 2014 | Sweden | Q | 1, bluelight.org, drugs-forum.com, and legalhighsforum.com. | unspecified | NR | NR | 1 | 3, Tong et al.(2013) | 1 | NR | 60 | 13082 | 2 | None | Government |
| Sowles 2018 | United States | Q with FA | 1, large Subreddit of Reddit | ED | NR | NR | 5 | own coding scheme for interaction | 3 | 28 | 16 | 240 | 2 | None | Government |
| Struik 2014 | Canada | Q with FA | 3, Crush the Crave app/Facebook page | ND | NR (18-34) | 44 | 2, 3 | NR | 1 | 7282 | 121 | 278 | 3 | None | Government |
| Yip 2019 | Hong Kong | Q with FA | 4, AnxietyCentral, Beyond Blue, Daily Strength, Mental Health Forum, No More Panic, PsyCentral | depression; anxiety | NR | NR | 5; 6; 10, sequence/paths analysis | Cutrona and Suhr; Bales’s Interaction Process Analysis | 2 | NR | 120 | 2036 | 2 | NR | NR |
| Walstrom 2000 | United States | Q | 4, USENET forum | anorexia, bulimia | NR | 100 | 7 | Cutrona and Suhr, modified by Coulson, own coding scheme for interaction | NR | NR | 10 | 4 | 3 | NR | NR |
| Worley 2019 | United States | Q with FA | 1, Bluelight (Sober Living) | substance abuse | NR | NR | 1 | NR | 1 | 148 | NR | NR | 2 | NR | NR |
| NR = not reported, PTSD = posttraumatic stress disorder; MCI = Mild Cognitive Impairment; BED = Binge Eating Disorder; CUD = cannabis use disorder; ADHD = attention deficit hyperactivity disorder; OCD = obsessive-compulsive disorder; ED = eating disorders; PPD = postpartum depression; GA = gambling addiction; ND = nicotine dependence; Q = qualitative; FA = frequency analysis; LDA = Latent Dirichlet Allocation, OUD = opioid use disorder  Type of analysis used was coded as follows: 1 = thematic analysis; 2 = framework; 3 = constant comparison; 4 = analytical induction; 5 = content analysis; 6 = conversational analysis; 7 = discourse analysis; 8 = interpretative phenomenological analysis; 9 = narrative analysis; 10 = other  * Bluelight, Cafe Pharma, Drug Forum, Addiction Treatment Watchdog, Drugs.com, Suboxone, the Light at the End of the Tunnel, MD Junction, Med Help, All Nurses.com, Pharmer, Prescription Drug-Info, Totse, Suboxone Zone, Zoklet  $ Cannabis addiction – Drugs-ForumCannabis addiction – Drugs-Forum, Online Rehab Group - CannabisRehab.org, Marijuana Anonymous: MA  & https://www.reddit.com/r/depression, www.depression-understood.org, www.psychforums.com/depression, healthunlocked.com, www.mentalhealthforum.net, www.alonelylife.com, psychcentralforums.com, www.dealingwithdepression.co.uk, www.beyondblue.org.au, www.depressionforums.org, www.beatingthebeast.com. | | | | | | | | | | | | | | | |
