# Supplementary material for: Meeting the Unmet Needs of Individuals With Mental Disorders: Scoping Review on Peer-to-Peer Web-Based Interactions
Source: JMIR Ment Health. 2022 Dec 5;9(12):e36056. doi: 10.2196/36056 (PMC9788841; doi:10.2196/36056)
Supplement: Multimedia Appendix 8 [file mental_v9i12e36056_app8.docx]

**This is a Multimedia Appendix to a full manuscript published in the JMIR Mental Health. For full copyright and citation information see** [**http://dx.doi.org/10.2196/36056**](http://dx.doi.org/10.2196/36056)

Table with means of codes per category and standard deviations for analyzed models

| ID | Mean (SD) | Ranking |
| --- | --- | --- |
| Model #1 (Cutrona) | 113.00 (128.95) | 13 |
| Model #2 (Cutrona modified) | 93.67 (82.38) | 10 |
| Model #3 (Rime) | 80.71 (54.36) | 4 |
| Model #4 (Gaysynsky) | 70.63 (108.94) | 12 |
| Model #5 (adapted by Liu) | 70.63 (69.65) | 6 |
| Model #6 (Wang) | 62.78 (70.47) | 7 |
| Model #7 (Bales) | 56.50 (73.12) | 8 |
| Model #8 (modified Greiner) | 43.46 (37.83) | **2** |
| Model #9 | 62.78 (46.55) | **3** |
| Model #10 | 70.63 (73.29) | 9 |
| Model #11 | 113.00 (88.56) | 11 |
| Model #12 | 188.33 (156.76) | 14 |
| Model #13 | 80.71 (67.96) | 5 |
| Model #14 | 37.67 (9.32) | **1** |
