# Supplementary material for: Meeting the Unmet Needs of Individuals With Mental Disorders: Scoping Review on Peer-to-Peer Web-Based Interactions
Source: JMIR Ment Health. 2022 Dec 5;9(12):e36056. doi: 10.2196/36056 (PMC9788841; doi:10.2196/36056)

**This is a Multimedia Appendix to a full manuscript published in the JMIR Mental Health. For full copyright and citation information see** [**http://dx.doi.org/10.2196/36056**](http://dx.doi.org/10.2196/36056)

Machine-learning results with confusion matrices

| ML technique | Parameter name | Parameter value | Accuracy [%] |
| --- | --- | --- | --- |
| Decision tree | min_samples_leaf | 1 | 81.03 |
| Decision tree | min_samples_leaf | 2 | 63.79 |
| Decision tree | min_samples_leaf | 3 | 55.17 |
| Logistic Regression | default | | 74.13 |
| Support Vector Machine | default | | 55.17 |
| k-Nearest Neighbours | n_neighbors | 2 | 50.00 |
| k-Nearest Neighbours | n_neighbors | 3 | 44.83 |
| k-Nearest Neighbours | n_neighbors | 4 | 44.83 |
| k-Nearest Neighbours | n_neighbors | 5 | 43.10 |
| Gaussian Naive Bayes | default | | 62.07 |

Not specified parameters were set to default.

**Confusion matrices**

ED = eating disorders; DEP = depression; PSU = psychoactive substance use; PPD = postpartum depression; AXD = anxiety disorders; PTSD = posttraumatic stress disorder; ADHD = attention deficit hyperactivity disorder; BD = bipolar affective disorder; MCI = Mild Cognitive Impairment OCD = obsessive-compulsive disorder; SZA = schizoaffective disorders; SCZ = schizophrenia

Decision tree, min_samples_leaf=1


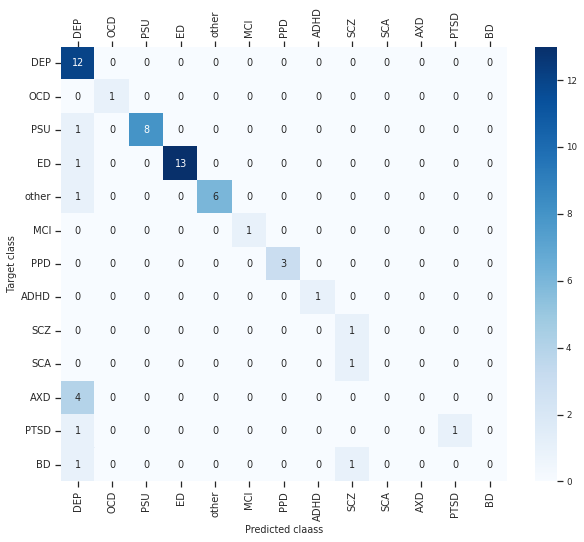


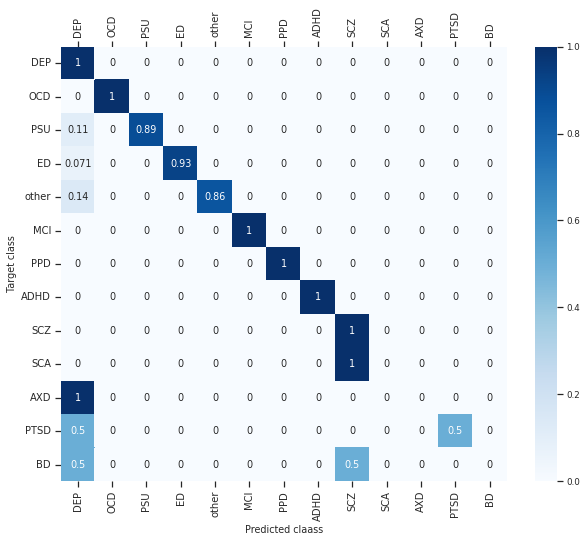


Decision tree, min_samples_leaf=2


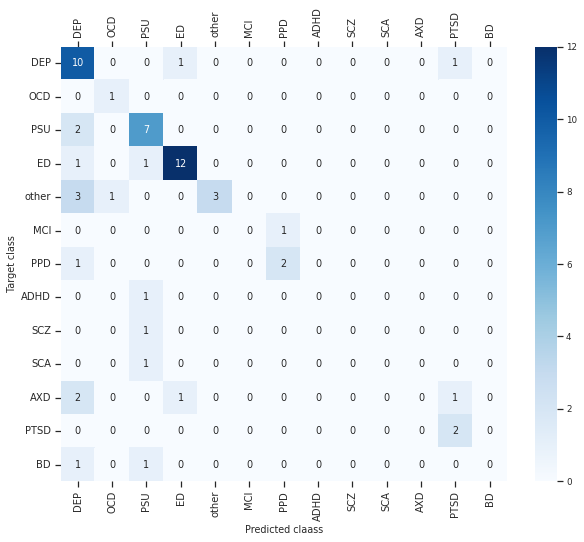


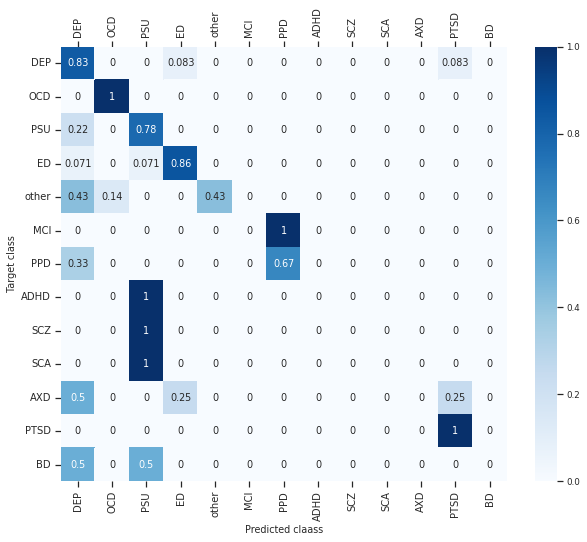


Decision tree, min_samples_leaf=3


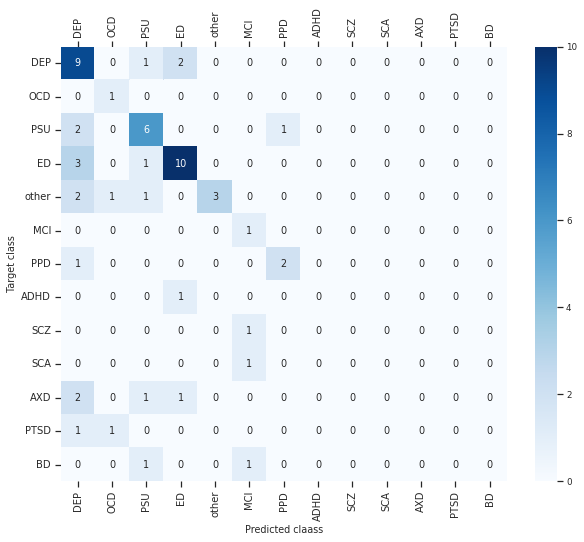


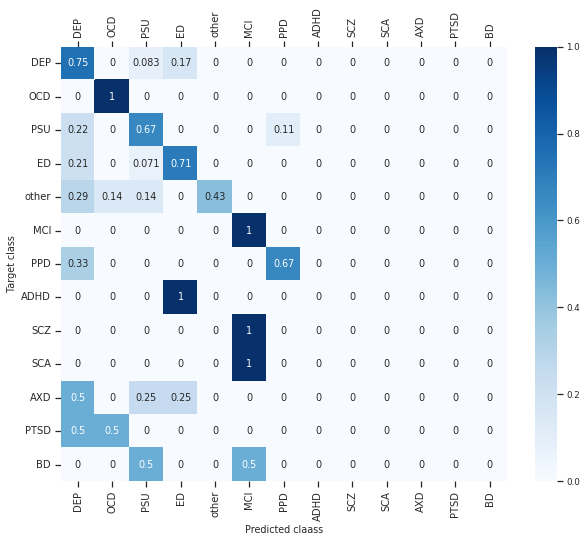


logistic regression


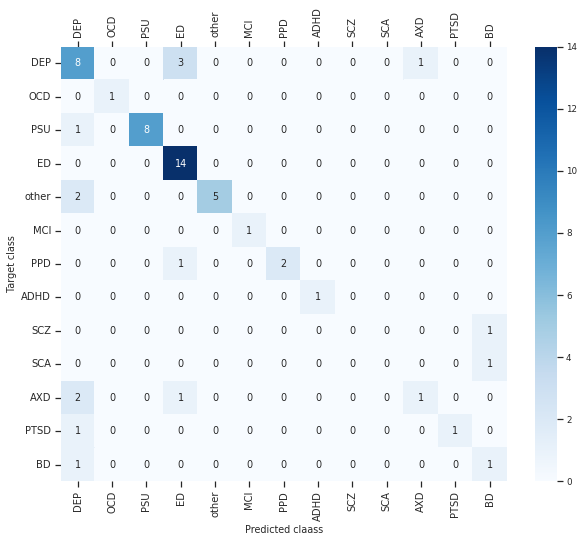


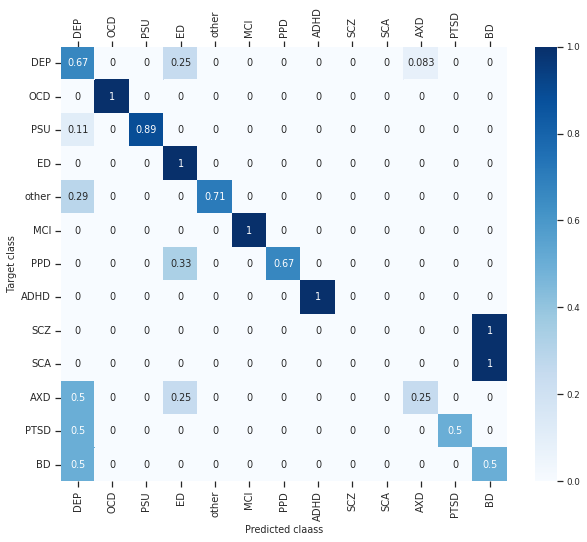


SVM


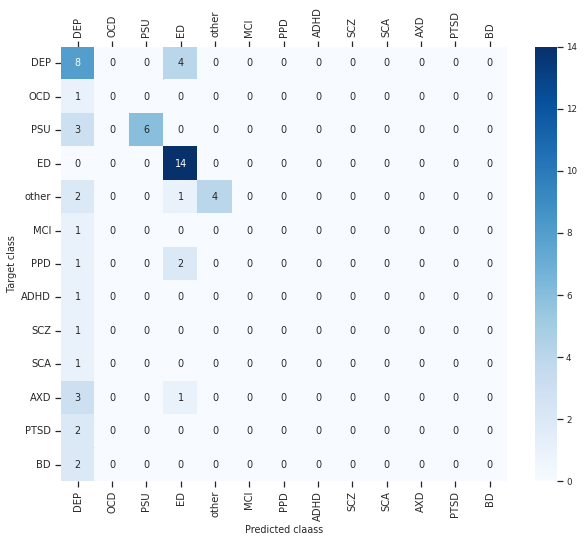


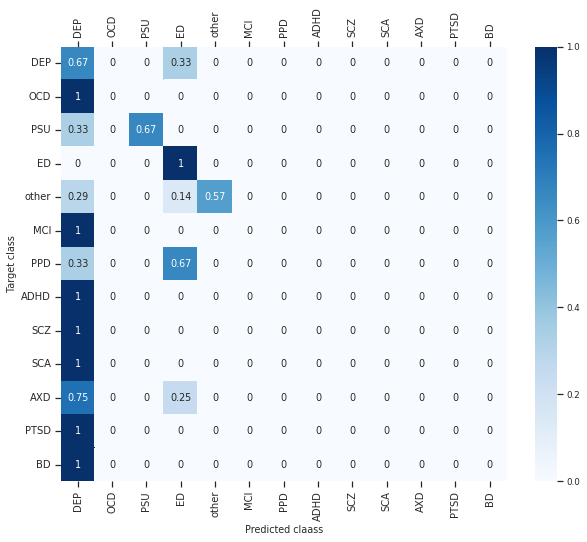


knn, n=2


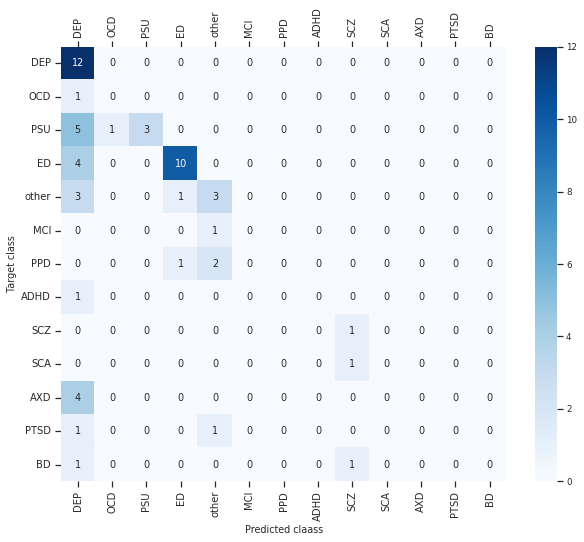


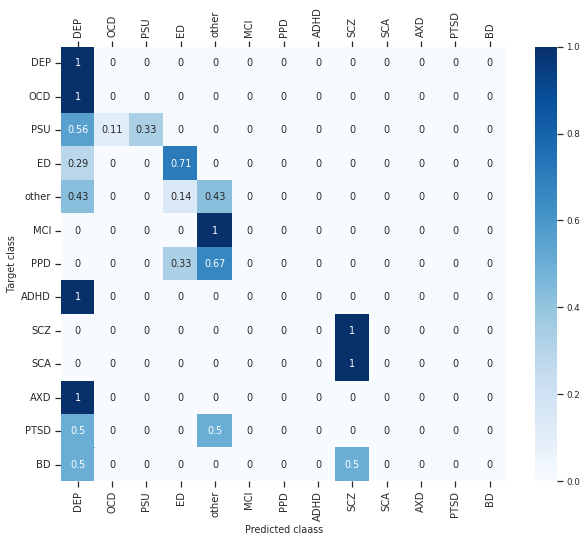


knn, n=3


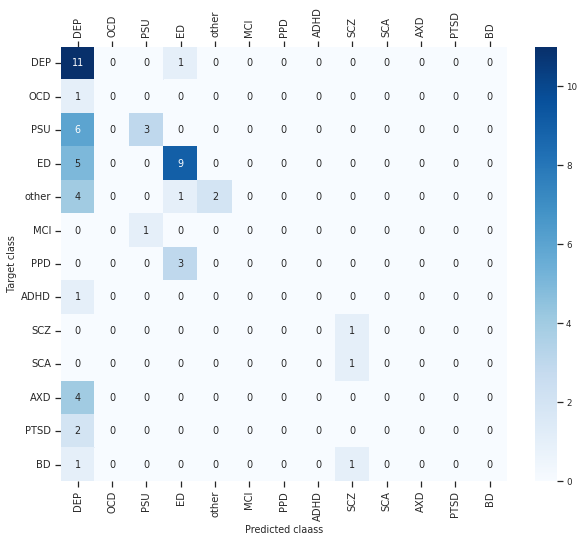


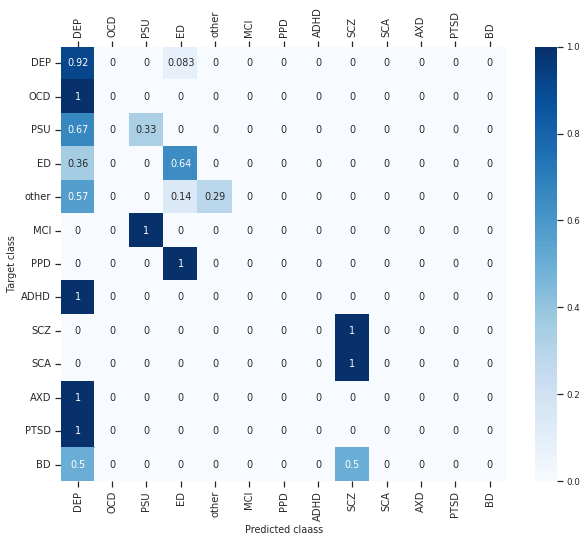


knn, n=4


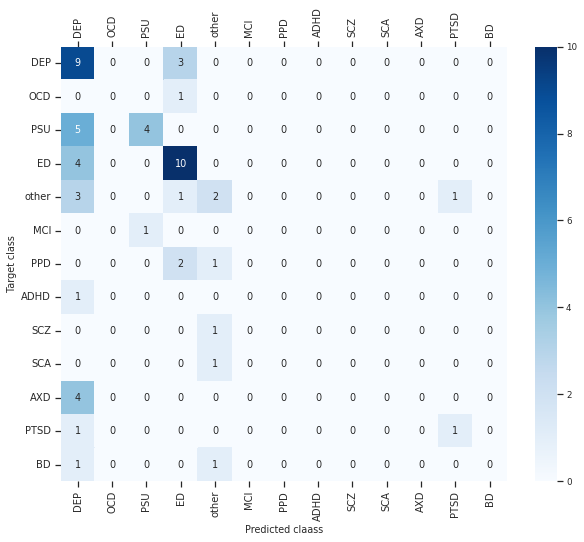


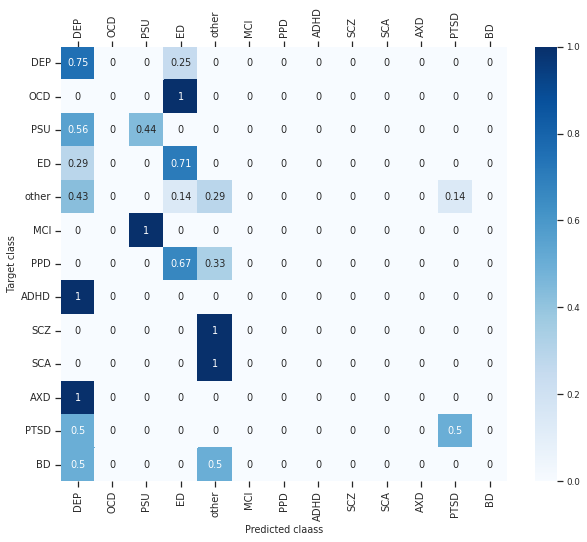


knn, n=5


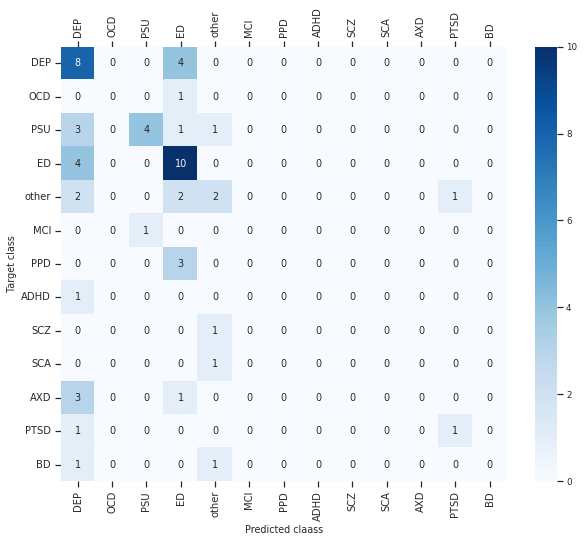


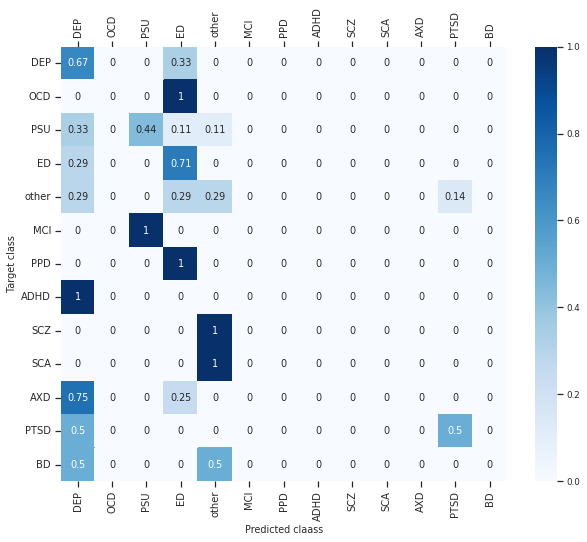


naive bayes


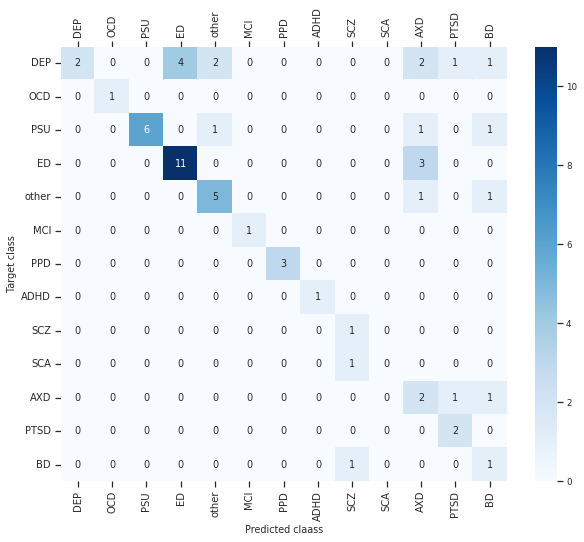


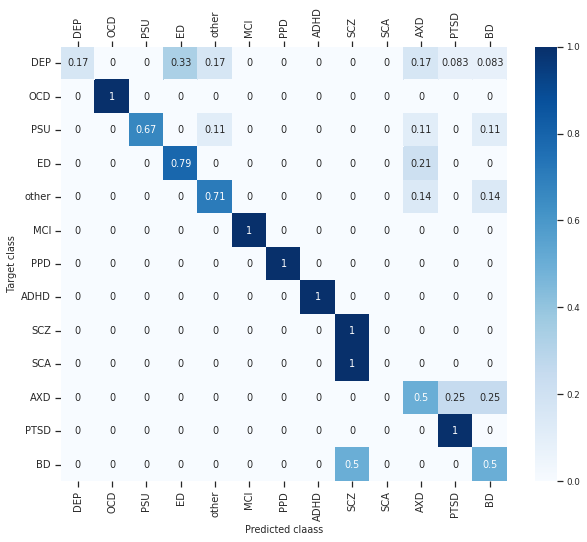

Supplement: Multimedia Appendix 11 [file mental_v9i12e36056_app11.docx]
