# Supplementary material for: Meeting the Unmet Needs of Individuals With Mental Disorders: Scoping Review on Peer-to-Peer Web-Based Interactions
Source: JMIR Ment Health. 2022 Dec 5;9(12):e36056. doi: 10.2196/36056 (PMC9788841; doi:10.2196/36056)
Supplement: Multimedia Appendix 12 [file mental_v9i12e36056_app12.docx]

**This is a Multimedia Appendix to a full manuscript published in the JMIR Mental Health. For full copyright and citation information see** [**http://dx.doi.org/10.2196/36056**](http://dx.doi.org/10.2196/36056)

Decision trees of prediction of diseases based on the presence of interaction

a) The minimum number of samples required to be at a leaf node: min_samples_leaf = 1


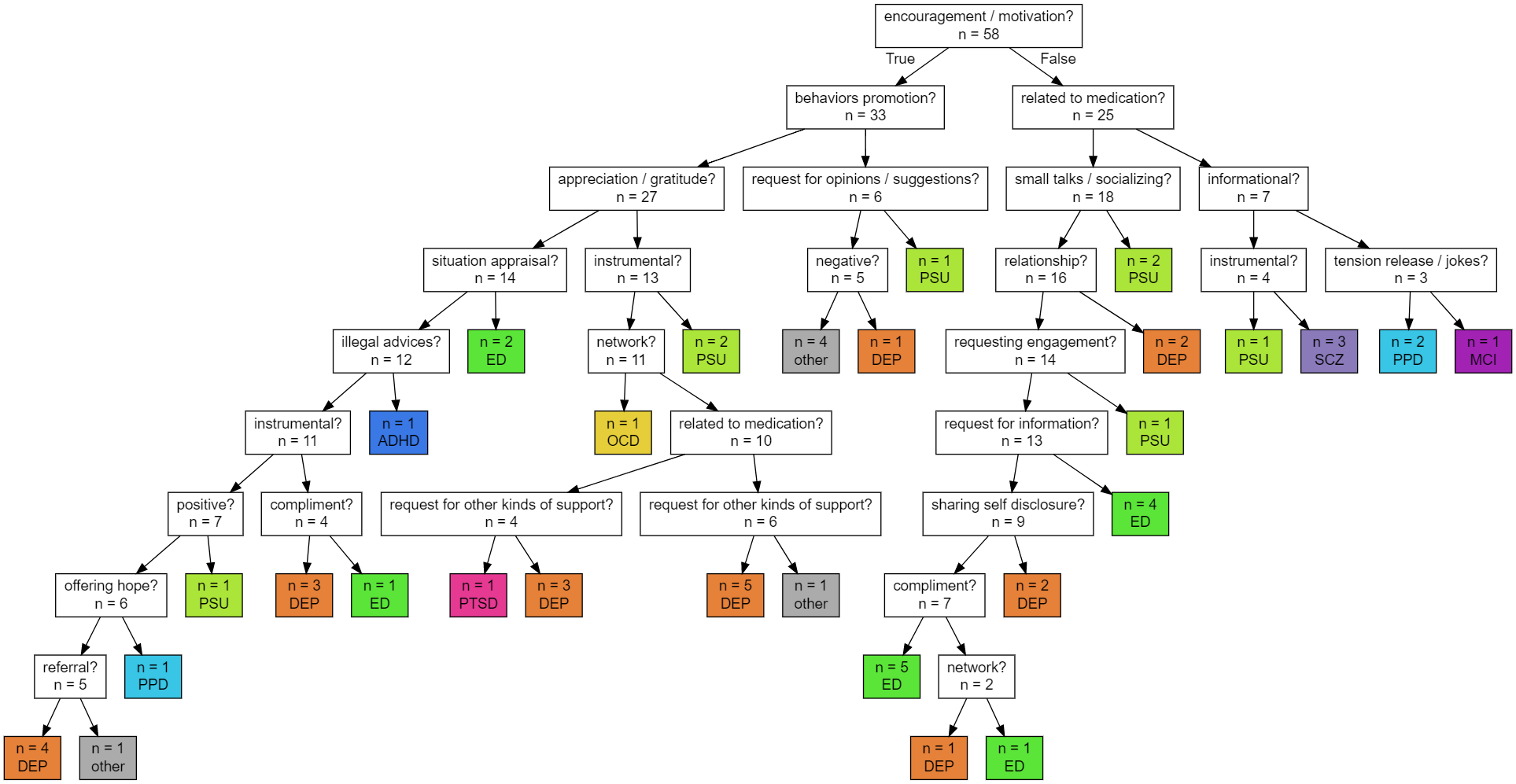


ED = eating disorders; DEP = depression; PSU = psychoactive substance use; PPD = postpartum depression; PTSD = posttraumatic stress disorder; ADHD = attention deficit hyperactivity disorder; MCI = mild cognitive impairment; OCD = obsessive-compulsive disorder; SCZ = schizophrenia.

b) The minimum number of samples required to be at a leaf node: min_samples_leaf = 2


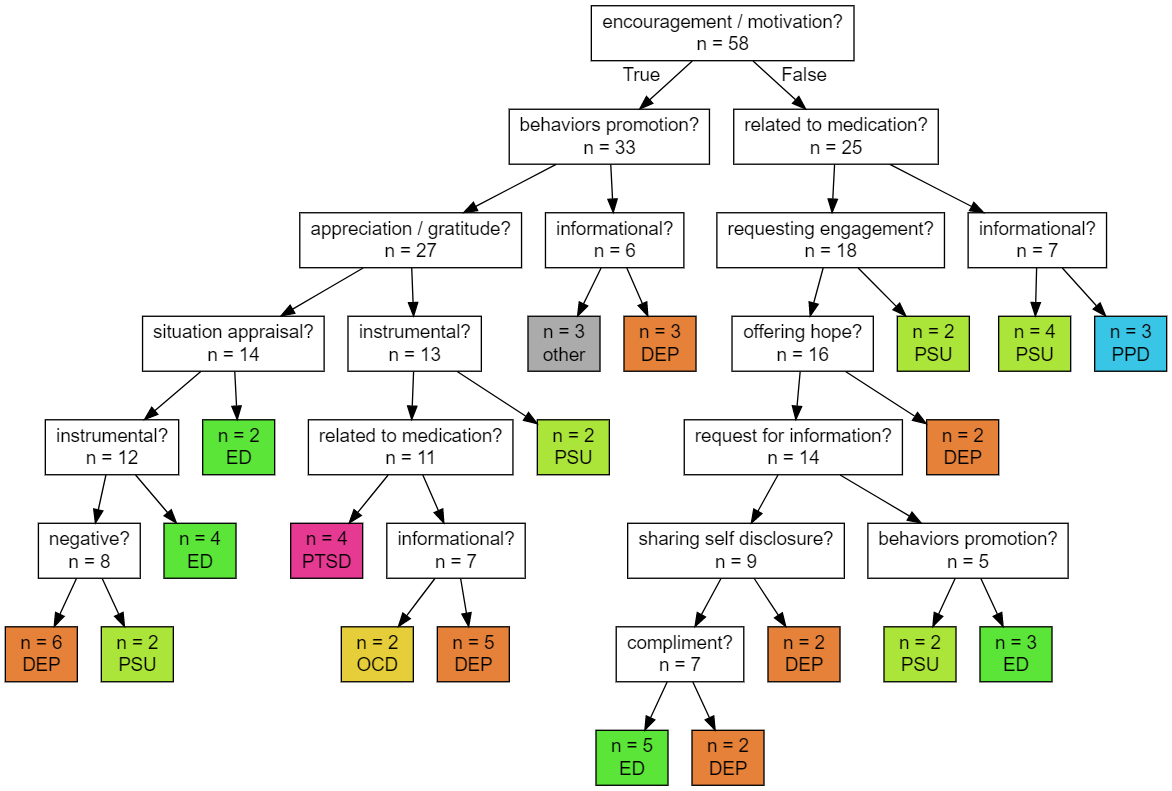


MCI = mild cognitive impairment; ADHD = attention deficit hyperactivity disorder, OCD = obsessive-compulsive disorder, ED = eating disorders; PPD = postpartum depression

c) The minimum number of samples required to be at a leaf node: min_samples_leaf = 3


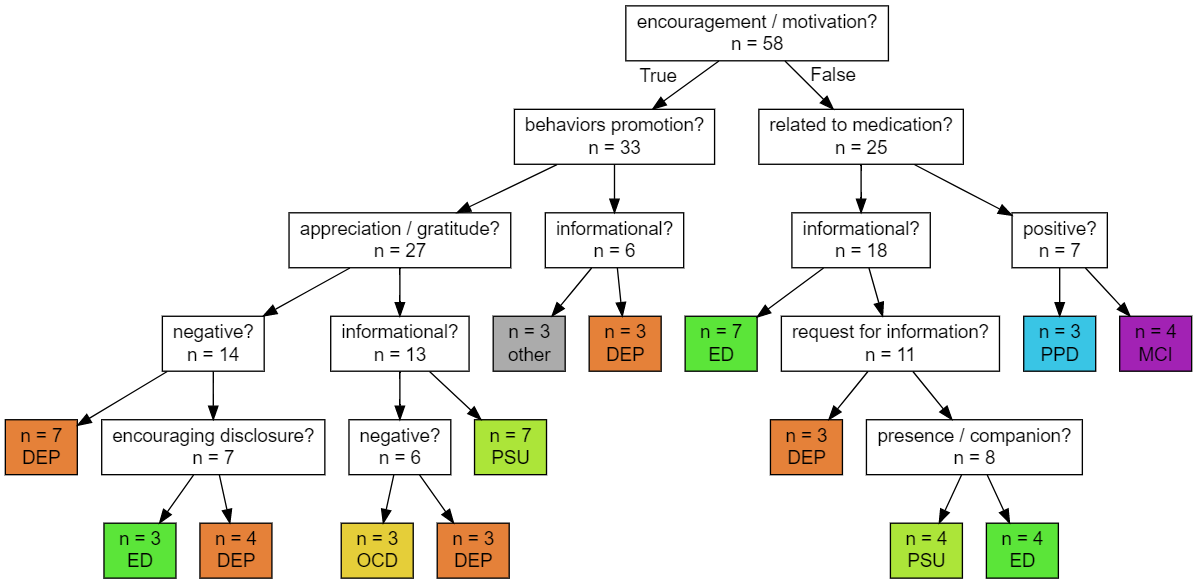


MCI = mild cognitive impairment; ADHD = attention deficit hyperactivity disorder, OCD = obsessive-compulsive disorder, ED = eating disorders; PPD = postpartum depression
