# Supplementary material for: Meeting the Unmet Needs of Individuals With Mental Disorders: Scoping Review on Peer-to-Peer Web-Based Interactions
Source: JMIR Ment Health. 2022 Dec 5;9(12):e36056. doi: 10.2196/36056 (PMC9788841; doi:10.2196/36056)

**This is a Multimedia Appendix to a full manuscript published in the JMIR Mental Health. For full copyright and citation information see** [**http://dx.doi.org/10.2196/36056**](http://dx.doi.org/10.2196/36056)

Quality of included studies


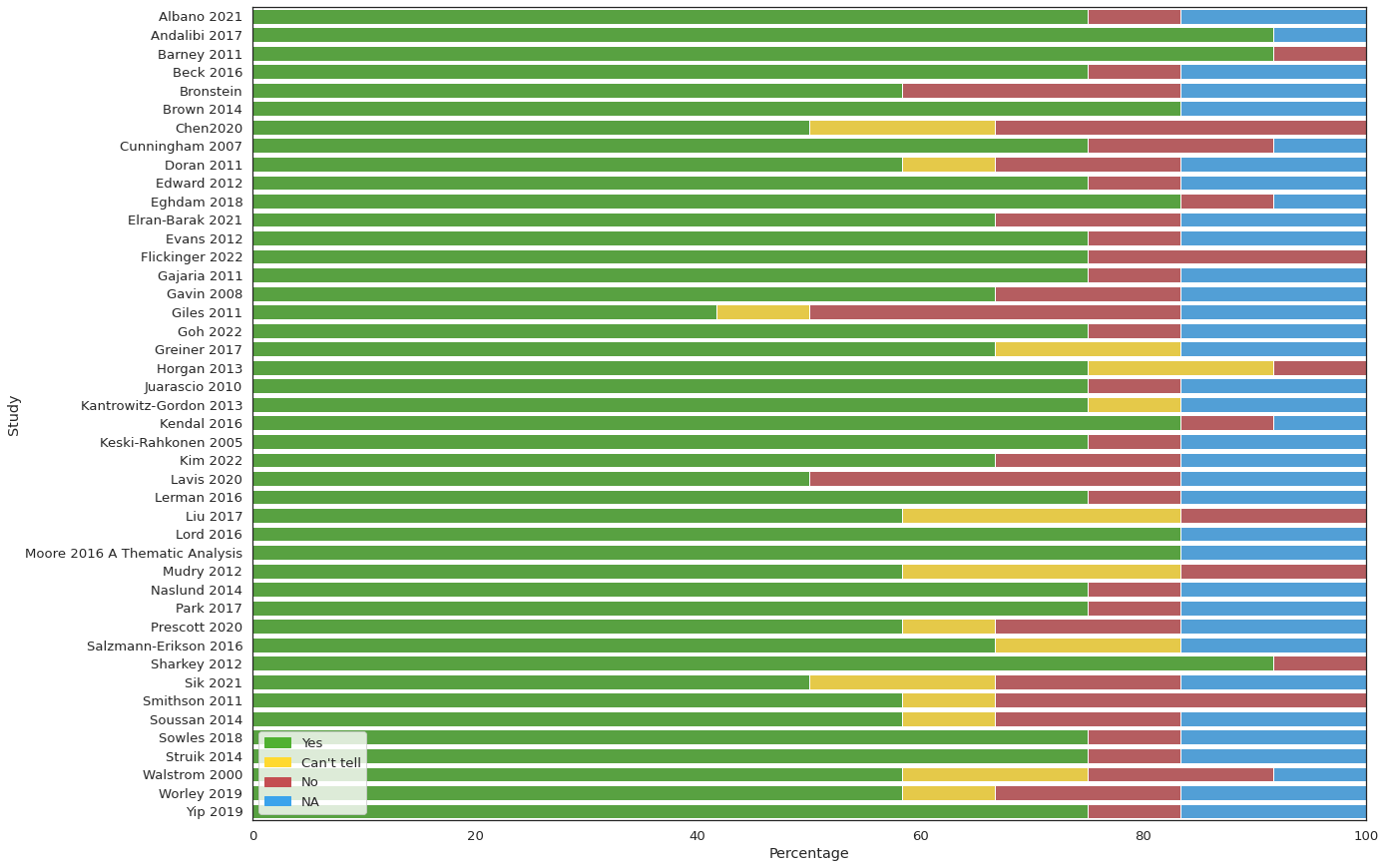

Supplement: Multimedia Appendix 13 [file mental_v9i12e36056_app13.docx]
