# Supplementary material for: Meeting the Unmet Needs of Individuals With Mental Disorders: Scoping Review on Peer-to-Peer Web-Based Interactions
Source: JMIR Ment Health. 2022 Dec 5;9(12):e36056. doi: 10.2196/36056 (PMC9788841; doi:10.2196/36056)
Supplement: Multimedia Appendix 14 [file mental_v9i12e36056_app14.docx]

**This is a Multimedia Appendix to a full manuscript published in the JMIR Mental Health. For full copyright and citation information see** [**http://dx.doi.org/10.2196/36056**](http://dx.doi.org/10.2196/36056)

List of clusters and related terms from VOSviewer

| Terms | Cluster | Occurrences | Terms | Cluster | Occurrences |
| --- | --- | --- | --- | --- | --- |
| supportive message | 3 | 8 | support category | 3 | 9 |
| supportive reply | 3 | 7 | support exchange | 3 | 5 |
| sympathy | 3 | 7 | support provider | 3 | 14 |
| tangible assistance | 3 | 9 | support seeker | 3 | 18 |
| tangible support | 3 | 5 | support solicitation | 3 | 17 |
| advice | 4 | 217 | support type | 3 | 17 |
| avoidance | 4 | 16 | care | 1 | 57 |
| support group participant status | 4 | 11 | communicative context | 4 | 11 |
| support group participation | 4 | 5 | competition | 4 | 7 |
| support group resource | 4 | 22 | disorder support group | 4 | 17 |
| supportive interaction | 4 | 8 | explanation | 4 | 39 |
| availability | 5 | 22 | f2f eating disorder support group | 4 | 38 |
| awareness | 5 | 31 | f2f support group | 4 | 8 |
| better understanding | 5 | 6 | question | 4 | 126 |
| membership categorization | 1 | 5 | solidarity | 4 | 59 |
| education | 1 | 29 | belief | 5 | 23 |
| expertise | 1 | 16 | experienced effect | 5 | 6 |
| exploration | 1 | 17 | group norm | 5 | 6 |
| financial support | 1 | 6 | illegal drug | 5 | 5 |
| health promotion | 1 | 9 | illicit drug | 5 | 7 |
| informal support | 1 | 5 | negative interaction | 5 | 7 |
| internet support group | 1 | 8 | self treat | 5 | 5 |
| online community | 1 | 56 | self treatment | 5 | 17 |
| online depression support forum | 1 | 6 | tolerance | 5 | 7 |
| online social network | 1 | 6 | trick | 5 | 7 |
| online social networking | 1 | 5 | trust | 5 | 15 |
| online social support | 1 | 19 | asking | 6 | 5 |
| online social support forum | 1 | 5 | community | 6 | 201 |
| online support | 1 | 43 | support staff | 1 | 13 |
| online support forum | 1 | 15 | discrimination | 6 | 8 |
| pregnancy | 1 | 30 | mental health community | 6 | 9 |
| privacy | 1 | 20 | need help | 6 | 7 |
| social | 1 | 17 | network | 6 | 70 |
| social network site | 1 | 7 | similarity | 6 | 38 |
| social relationship | 1 | 8 | social network analysis | 6 | 16 |
| unsolicited advice | 1 | 6 | validity | 6 | 19 |
| virtual community | 1 | 10 | acceptance | 7 | 42 |
| therapeutic potential | 1 | 8 | anonymity | 7 | 70 |
| blame | 2 | 9 | authenticity | 7 | 9 |
| confession | 2 | 39 | community member | 7 | 11 |
| congratulation | 2 | 6 | confidentiality | 7 | 10 |
| encouragement | 2 | 50 | criticism | 7 | 10 |
| experience | 2 | 372 | group identity | 7 | 19 |
| explicit question | 2 | 24 | identification | 7 | 21 |
| good luck | 2 | 11 | identity | 7 | 162 |
| greeting | 2 | 8 | joke | 7 | 10 |
| help | 2 | 339 | mutual support | 7 | 8 |
| hope | 2 | 53 | social norm | 7 | 6 |
| hopelessness | 2 | 7 | support forum | 7 | 25 |
| hug | 2 | 12 | understanding | 7 | 100 |
| motivator | 9 | 11 | communication support | 8 | 25 |
| social networking | 9 | 16 | little help | 8 | 17 |
| social networking site | 9 | 41 | negative response | 8 | 8 |
| unhealthy coping behavior | 9 | 5 | secrecy | 10 | 16 |
| validation | 9 | 53 | positive social support | 8 | 13 |
| assistance | 10 | 27 | information | 10 | 576 |
| emotional expression | 10 | 16 | online support group | 10 | 138 |
| prayer | 2 | 11 | information need | 10 | 24 |
| relationship | 2 | 127 | professional support | 10 | 5 |
| rule | 2 | 28 | secret | 10 | 14 |
| secretway | 2 | 6 | useful information | 10 | 6 |
| shame | 2 | 37 | usefulness | 10 | 5 |
| side effect | 2 | 24 | disclosure | 11 | 158 |
| socialization | 2 | 7 | exchange | 11 | 67 |
| supporter | 2 | 6 | group cohesion | 11 | 9 |
| thank | 2 | 33 | instruction | 11 | 8 |
| warning | 2 | 10 | professional treatment | 11 | 9 |
| agreement | 3 | 17 | reluctance | 11 | 11 |
| asks | 3 | 8 | social network | 11 | 34 |
| clarification | 3 | 9 | understanding depression | 11 | 5 |
| companionship | 3 | 13 | withdrawal | 11 | 12 |
| companionship support | 3 | 14 | engagement | 12 | 34 |
| compliment | 3 | 11 | support group | 12 | 155 |
| confidence | 3 | 16 | support network | 12 | 13 |
| direct request | 3 | 28 | mental health support | 13 | 9 |
| direct support request | 3 | 6 | online peer support | 13 | 11 |
| emotional | 3 | 9 | professional help | 13 | 42 |
| emotional disclosure | 3 | 17 | emphasis | 14 | 12 |
| emotional distress | 3 | 7 | informal peer support | 14 | 5 |
| emotional support | 3 | 174 | negative comment | 14 | 12 |
| empathy | 3 | 43 | peer exchange | 14 | 5 |
| esteem | 3 | 9 | peer support | 14 | 86 |
| esteem support | 3 | 11 | friendship | 15 | 30 |
| friendly message | 3 | 5 | helpseeking | 15 | 6 |
| gratitude | 3 | 80 | selfdisclosure | 3 | 12 |
| gratitude expression | 3 | 20 | shows solidarity | 3 | 8 |
| group interaction | 3 | 14 | social support | 3 | 342 |
| indirect request | 3 | 10 | social support category | 3 | 10 |
| information sharing | 3 | 5 | social support exchange | 3 | 6 |
| informational support | 3 | 90 | social support group | 3 | 10 |
| instrumental support | 3 | 19 | social support type | 3 | 12 |
| negative emotional disclosure | 3 | 6 | support | 3 | 629 |
| network support | 3 | 22 | request | 3 | 73 |
| online depression support group | 3 | 7 | self disclosure | 3 | 103 |
